# Crosstalk Between Pheromone Signaling and NADPH Oxidase Complexes Coordinates Fungal Developmental Processes

**DOI:** 10.3389/fmicb.2020.01722

**Published:** 2020-07-28

**Authors:** Sarah Schmidt, Ramona Märker, Barbara Ramšak, Anna M. Beier-Rosberger, Ines Teichert, Ulrich Kück

**Affiliations:** Allgemeine und Molekulare Botanik, Ruhr-Universität Bochum, Bochum, Germany

**Keywords:** pheromone response pathway, nicotinamide adenine dinucleotide phosphate oxidase complexes, sexual development, ascospore germination, *Sordaria macrospora*

## Abstract

Sexual and asexual development in filamentous ascomycetes is controlled by components of conserved signaling pathways. Here, we investigated the development of mutant strains lacking genes for kinases MAK2, MEK2, and MIK2, as well as the scaffold protein HAM5 of the pheromone response (PR) pathway. All had a defect in fruiting body development and hyphal fusion. Another phenotype was a defect in melanin-dependent ascospore germination. However, this deficiency was observed only in kinase deletion mutants, but not in strains lacking HAM5. Notably, the same developmental phenotypes were previously described for nicotinamide adenine dinucleotide phosphate (NADPH) oxidase 1 (NOX1) mutants, but the germination defect was only seen in NOX2 mutants. These data suggest a molecular link between the pheromone signaling pathway and both NOX complexes. Using data from yeast two-hybrid (Y2H) analysis, we found that the scaffolding protein HAM5 interacts with NOR1, the regulator of NOX1 and NOX2 complexes. This interaction was further confirmed using differently fluorescent-labeled proteins to demonstrate that NOR1 and HAM5 co-localize at cytoplasmic spots and tips of mature hyphae. This observation was supported by phenotypic characterization of single and double mutants. The oxidative stress response and the initiation of fruiting bodies were similar in Δham5Δnor1 and Δham5, but distinctly reduced in Δnor1, indicating that the double deletion leads to a partial suppression of the Δnor1 phenotype. We conclude that the PR and NOX1 complexes are connected by direct interaction between HAM5 and NOR1. In contrast, PR kinases are linked to the NOX2 complex without participation of HAM5.

## Introduction

Multicellular development and differentiation are highly complex processes orchestrated by the interaction of several tightly regulated signaling modules. In filamentous fungi, these processes are governed by several highly conserved signaling modules, including mitogen-activated protein kinase (MAPK) pathways, such as the pheromone response (PR) pathway and cell wall integrity (CWI) pathway, two nicotinamide adenine dinucleotide phosphate (NADPH) oxidase (NOX1 and NOX2) complexes, and the striatin-interacting phosphatases and kinases (STRIPAK) complex (Pöggeler et al., 2018; Riquelme et al., 2018; Fischer and Glass, 2019).

MAPK pathways comprise three-tiered kinase cascades, which sequentially activate each other through phosphorylation. The activated downstream kinase subsequently phosphorylates many target proteins, including transcription factors (Martínez-Soto and Ruiz-Herrera, 2017). NOX complexes generate reactive oxygen species (ROS), which are also important players in both intracellular and extracellular signaling (Lara-Ortíz et al., 2003; Scott and Eaton, 2008; Aguirre and Lambeth, 2010). The STRIPAK complex regulates many other proteins and cascades by regulating their phosphorylation status (Märker et al., 2020).

Recently, investigations into several fungal developmental processes have suggested an interplay between these pathways. For example, all of these pathways are involved in the regulation of germling and hyphal fusion in *Neurospora crassa*. Germling and hyphal fusion are regulated by strict spatiotemporal dynamics of the MAPK pathways, leading to an antiphase oscillation of PR pathway components and the CWI scaffold SOFT at germling and hyphal tips (Fischer and Glass, 2019). Furthermore, it has been shown that phosphorylation of the CWI pathway components during germling fusion is dependent on the PR pathway in *N. crassa* (Fu et al., 2014).

Another example of interplay is the nuclear translocation of MAK1, the downstream kinase of the CWI pathway, which is dependent on the STRIPAK subunit MOB-3 in vegetative hyphae. The requirement of PR-dependent phosphorylation of MOB-3 during this process shows the importance of crosstalk between different signaling pathways (Dettmann et al., 2013). In the filamentous ascomycete *Podospora anserina*, NOX1 is required for nuclear accumulation of the MAPK from the CWI pathway (Kicka et al., 2006), and in the rice blast fungus *Magnaporthe oryzae*, components of the PR pathway and the NOX2 complex are required for proper development of appressorium-like structures during plant infection (Breitenbach et al., 2015; Yan and Talbot, 2016; Jiang et al., 2018). Furthermore, a link between the NOX2 complex and the PR pathway was observed in *P. anserina* during ascospore germination, since mutants carrying gene deletions of either the NOX2 or PR module were defective in germination of melanized ascospores (Brun et al., 2009; Lalucque et al., 2012).

Here, we used the filamentous fungus *Sordaria macrospora* to demonstrate crosstalk between the PR pathway and the NOX complexes during the development. *S. macrospora* is a eukaryotic model organism for sexual multicellular development since it lacks any vegetative propagation structures, such as conidiospores (Teichert et al., 2014a). Since this fungus is self-fertile, the sexual cycle is completed without mating between strains. Thus, any developmental defect in fruiting body formation can be detected directly. A collection of sterile mutants recently enabled the isolation of genes required for the formation of fruiting bodies (Kück et al., 2009; Engh et al., 2010). The genes identified encoded components of the CWI pathway, the NOX1 complex, and the STRIPAK complex (Bloemendal et al., 2010, 2012; Dirschnabel et al., 2014; Teichert et al., 2014b; Nordzieke et al., 2015; Beier et al., 2016).

In this study, we analyzed the function of components of the PR pathway. Kinases of this signaling complex have already been shown to be involved in the sexual cycle of diverse ascomycetes (Bayram et al., 2012; Lalucque et al., 2012; Lichius et al., 2012; Frawley et al., 2018). Here, we investigated PR-dependent fruiting body formation, cell fusion, and ascospore germination. Furthermore, we investigated the cellular localization of PR components using fluorescence microscopy. Our study was extended by yeast two-hybrid (Y2H) experiments, co-localization, and genetic studies to demonstrate that during distinct stages of the life cycle, the kinases of the PR pathway interact differently with either of two NOX complexes. We suggest that HAM5, the scaffolding protein of the PR complex, mediates crosstalk with the NOX1, but not with the NOX2 module.

## Materials and Methods

### Strains and Growth Conditions

*Escherichia coli* strain XL1 Blue MRF' (Jerpseth et al., 1992) was used as a host for cloning and propagation of recombinant plasmids under standard laboratory conditions (Sambrook and Russell, 2001). Alternatively, *Saccharomyces cerevisiae* PJ69-4A (James et al., 1996) was used for plasmid construction *via* homologous recombination, as described by Colot et al. (2006), and recombinant yeast strains were selected by prototrophy to uracil. Yeast experiments were carried out according to standard protocols (Clontech Yeast Protocol Handbook, PT3024-1).

Details for all *S. macrospora* strains used in this study are given in Supplementary Table S1. The wild type strain (R19027) and the spore color mutant fus (S70823) were obtained from our laboratory collection. Standard laboratory conditions were used for culturing *S. macrospora* strains (Kamerewerd et al., 2008; Dirschnabel et al., 2014), unless indicated otherwise. DNA-mediated transformation was performed as described by Nordzieke et al. (2015), but excluding the treatment with caylase. Subsequently, transformants were selected using solid medium containing hygromycin B (80 U/ml) and/or nourseothricin (100 μg/ml). Growth and stress tests were conducted three times for each strain analyzed using three technical replicates in each experiment. After pre-culturing the strains on corn meal-malt fructification medium (BMM) for 4 days, a standardized piece of the pre-culture was used to inoculate solid synthetic Westergaard’s medium (SWG). Mycelial growth was measured after 24 and 48 h. SWG medium contained 0.001% (v/v) H_2_O_2_ for oxidative stress assays.

### Generation of Deletion Strains

Plasmids pFlip5-MAK2 and pFlip3-MAK2 were generated for deletion of *mak2*. For pFlip5-MAK2, the 5' flank was amplified from *S. macrospora* genomic DNA using primers MAK2-KO1-KpnI/MAK2-KO2-SnaBI and ligated into pFlip (Bloemendal et al., 2014) using *Kpn*I/*Sna*BI. Primers MAK2-KO3-HindIII/MAK2-KO4-BglII were used for amplification of the 3' flank and ligated with *Hind*III/*Bgl*II into pFlip to generate pFlip3-MAK2.

pKO-MEK2 was generated by amplification of the 5' and 3' flanking regions from *S. macrospora* genomic DNA using primer pairs mek2_5_fw/mek2_5_rv and mek2_3_fw/mek2_3_rv. An *hph* cassette was amplified from pDrive-hyg (Engh et al., 2007b) using primers ptrpC-mek2/hph_rev. All fragments were cloned into *Eco*RI-restricted pDrive (QIAGEN, Hilden, Germany) using the In-Fusion HD Cloning Kit (Takara Bio, Saint-Germain-en-Laye, France).

Deletion vectors of *mik2* and *ham5* were generated by yeast recombination. For pKO-MIK2, 5' (1,000 bp) and 3' (1,000 bp) flanking regions of *mik2* were PCR-amplified using *S. macrospora* genomic DNA and primer pairs 5356-5fw/5356-5rv and 5356-3fw/5356-3rv, respectively. Flanking regions were transformed into yeast together with an *hph* cassette cut with *Eco*RI from plasmid pSF27-34 (Nowrousian and Cebula, 2005) and *Eco*RI-linearized vector pRS426 (Christianson et al., 1992).

For pKO-HAM5, the 3' and 5' flanks were amplified using *S. macrospora* genomic DNA and primer pairs 2471_5fw_neu/2471_5rv_neu and 2471_3fw/2471_3fw. The *hph* cassette was excised from vector pSF27-34, and all fragments were transformed into yeast together with *Eco*RI/*Xho*I-restricted pRS426.

Linearized pKO-MIK2, pKO-MEK2, pKO-MAK2, and pKO-HAM5 were transformed into *S. macrospora* Δku70 (Pöggeler and Kück, 2006). For removal of the *ku70* deletion, and to obtain homokaryotic strains derived from a single ascospore, strains were crossed with the spore color mutant fus. This mutation is contained in the genomes of most strains investigated in this study; however, for clarity reasons, this is not mentioned further in the figures, except when we used this mutation for measuring the frequencies of ascospore germination. The double deletion strain Δham5Δnor1/fus was obtained through crossing of Δham5 against Δnor1/fus. Gene deletion of all strains was verified using PCR analysis and Southern hybridization (Supplementary Figures S1–S5).

### Cloning Vectors for Complementation and Localization

For gfp-tagged versions of *mak2* and *mek2*, both were amplified from genomic DNA using primer pairs gfp_mak2_fw/gfp_mak2_rv and gfp_mek2_fw/gfp_mek2_rv, respectively. The resulting fragments of 1,325 and 1,733 bp were cloned into *Bam*HI-digested pDS23 using homologous recombination in yeast to generate pgfp-mak2 and pgfp-mek2.

Golden Gate cloning was used to generate gfp-tagged *mik2* and *ham5* vectors, as described by Marillonnet and Werner (2015). For complementation and localization, *mik2* was amplified from genomic DNA using primer pairs mik2.1_FWD/mik2.1_REV, mik2.2_FWD/mik2.2_REV, and mik2.3_FWD/mik2.3_REV. Fragments were subcloned into pJET1.2/blunt (Thermo Fisher Scientific, USA) and cloned into pGG-N-EGFP (Teichert, unpublished data) using the Golden Gate cloning procedure to generate pgfp-mik2.

pGG-C-EGFP-HAM5 was generated by amplification of *ham5* from gDNA using primers ham5-fw1/ham5-rv1 and ham5-fw2/ham5-rv3 and subsequent cloning into pGG-C-EGFP (Teichert, unpublished data) using the Golden Gate reaction. For pGG-N-EGFP-HAM5, primer pairs ham5-fw1/ham5-rv1 and ham5-fw2/ham5-rv4 were used to amplify *ham5* from pGG-C-EGFP-HAM5. Golden Gate cloning was used to insert both fragments of *ham5* into pGG-N-EGFP.

For plasmid pGG-Nor1-gfp, *nor1* was amplified from gDNA using primers GG_nor1_for/GG_nor1_rev and cloned into pGG-C-EGFP using Golden Gate cloning.

For pNor1-mCherry, *mCherry* was amplified from the plasmid pCherry using primers EcoRV-mCherry-for/1757. The fragment and pGG-Nor1-GFP were restricted using *Eco*RV/*Bam*HI. The fragment was subsequently ligated into the 7,202 bp fragment of pGG-Nor1-GFP.

For pH2A-mRFP, a 560 bp *Nco*I fragment from pYH2A (Rech et al., 2007) was cloned into the *Nco*I site of pMSHnat (Teichert, unpublished data).

To localize spindle pole bodies (SPBs), plasmids pGG-mRFP-GRC1 and pTUB4-mCherry were generated. For pGG-mRFP-GRC1, *grc1* was amplified from gDNA using primers GG_01693_for1/GG_01693_rev1 and GG_01693_for2/GG_01693_rev2. Fragments were integrated into pGG-N-mRFP (Teichert, unpublished data) using Golden Gate cloning. For pTUB4-mCherry, primers NotI-tub4-for/EcoRI-tub4-rev were used to amplify *tub4* from gDNA. Fragments were cut using *Not*I/*Eco*RI and ligated into *Not*I/*Eco*RI-digested pCherry.

All genes were integrated ectopically and expressed using a *gpd* promoter.

### Yeast Two-Hybrid Studies

All plasmids and oligonucleotides used in this study are listed in Supplementary Tables S2 and S3. For Y2H analyses, PCR was performed on *S. macrospora* cDNA, and PCR fragments were cloned into pGADT7 and pGBKT7 as follows:

For *mak2* vectors, PCR-fragments produced with primer pairs 3492-01_AD/3492-02_AD or 3492-01_BD/3492-02_BD were transformed into yeast together with *Sma*I-digested pGADT7 or pGBKDT to generate pA-3492 or pB-3492.

For *mek2* vectors, a 1,545 bp *mek2* cDNA fragment was amplified with primers 6526-01_Nde/6526-02_Bam and ligated *Nde*I/*Bam*HI into pGADT7 or pGBKT7 to generate pA-6526 or pB-6526.

To generate pA-5356, *mik2* was partly amplified from cDNA using primers 5356-01_Eco/5356-04 or 5356-03/5356-02_Bam and subcloned in pDrive to generate pDrive-5356a or pDrive-5356b. pDrive-5356a was digested with *Eco*RI, and the resulting fragment was ligated into *Eco*RI-restricted pGADT7 to generate pA-5356a. Subsequently, pDrive-5356b was digested using *Bam*HI/*Xba*I and ligated into *Bam*HI/*Xba*I-digested pA-5356a. pB-5356 was generated by ligation of a 2,751 bp *Eco*RI/*Bam*HI fragment from pA-5356 into *Eco*RI/*Bam*HI-digested pGBKT7.

For Y2H analysis of *ham5*, two fragments of *ham5* were amplified from *S. macrospora* cDNA or gDNA using primer pairs 2471_01AD/2471_02 and 2471_03/2471_06_BD1. Fragments were transformed into yeast together with *Eco*RI-restricted pGADT7 to generate pA-2471. For pB-2471, pA-2471 was digested with *Nde*I/*Bam*HI, and the resulting fragment was ligated into *Nde*I/*Bam*HI-restricted pGBKT7.

For *nor1* vectors, a 1,574 bp *nor1* fragment was amplified from cDNA using primers 2124_fw/2124_rv. The fragment was restricted with *Bam*HI and cloned into *Bam*HI-digested pGADT7 or pGBKT7 to generate pA-2124 or pB-2124.

pGADT7 and pGBKT7 derivatives were transformed by electroporation into yeast strains PJ69-4α and PJ69-4a, respectively, and transformants were mated to generate diploid strains as previously described (Becker and Lundblad, 1994; Kopke et al., 2013). To select for diploids, strains were plated on medium lacking leucine and tryptophan, while reporter gene activity was analyzed on medium lacking leucine, tryptophan, adenine, and histidine. Drop plating assays were performed as previously described (Teichert et al., 2014b).

### Quantification of Fruiting Body Formation

The initiation of fruiting body development was quantified after 3 days of incubation on BMM-covered microscope slides. Ascogonia and protoperithecia were counted separately within an area of 0.5 cm^2^ located 1 cm behind the growth front. An Axio Imager M1 (Zeiss) with a SpectraX LED lamp (Lumencor) and a Photometrix Cool SnapHQ camera (Roper Scientific, Martinsried, Germany) was used for quantification. Experiments were repeated for three biological replicats per strain. For assessment of fruiting body formation, images of strains were taken after 7 days of incubation in Petri dishes on solid BMM with a Zeiss Stemi 2000-C binocular, using an AxioCam ERc5s with the software ZEN 2 core (version 2.5, Zeiss, Jena, Germany).

### Ascospore Germination Assay

An ascospore germination assay was performed to quantitatively assess the germination capacity of black ascospores carrying distinct gene deletions. This assay was conducted by crossing deletion strains carrying the *fus1-1* mutation against the wild type strain. After 11 days, black and brown ascospores were isolated from recombinant perithecia and recovered on BMM-NaAc. The experiment was repeated until at least 100 ascospores of each spore color germinated from each cross. For strains with a generally decreased germination rate, only 50 germinated ascospores of each color were collected per cross. Germinated ascospores were tested for hygromycin resistance and ascospores from crosses of marker-free strains for sterility or fertility.

### Microscopic Investigation

Microscopic investigation of strains was conducted using either conventional or confocal light microscopy. The Axio Imager M1 microscope (Zeiss) with Metamorph software (version 7.7.0.0; Universal Imaging, Bedford Hills, NY, USA) was used for light and conventional fluorescence microscopy. For imaging of 4',6-diamidino-2-phenylindole (DAPI; Serva, Heidelberg, Germany) and Calcofluor White M2R (CFW; Sigma Aldrich, St. Louis, MO, USA) stainings, Chroma filter set (Chroma Technology Corp., Bellows Falls, VT, USA) 31000v2 (excitation filter D350/50, emission filter D460/50, and beam splitter 400dclp) were used. EGFP was detected using the filter set 49002 (excitation filter HQ470/40, emission filter HQ525/50, and beam splitter T495LPXR) and mRFP, as well as mCherry were detected with the filter set 49008 (excitation filter HQ560/40, emission filter ET630/75m, and beam splitter T585lp). Images were acquired using the SpectraX LED lamp (Lumencor) and the Photometrix Cool SnapHQ camera (Roper Scientific, Martinsried, Germany).

For microscopic investigation of sexual development and fluorescent imaging, strains were inoculated on BMM-covered slides and mounted with a coverslip using 0.7% sodium chloride (NaCl) solution. For assessment of sexual development, strains were grown for 3 (ascogonia, unpigmented protoperithecia), 5 (pigmented protoperithecia), and 7 days (perithecia). Fluorescent imaging of hyphae was conducted after 2 days of incubation. Nuclei were stained using DAPI in a concentration of 5 mg/ml diluted in 0.7% NaCl solution. Ascogonial septation was analyzed by staining of the cell wall using CFW. The 1 μg/ml CFW stock solution was diluted 1:400 in a 0.9% NaCl solution.

Hyphal fusion events were investigated by observing strains grown on a layer of cellophane on top of solid MMS medium for 2 days (Rech et al., 2007).

Confocal microscopy was carried out using a Leica TCS SP5 II microscope (Leica DMI6000 B, Wetzlar, Germany) with Leica Application Suite Advanced Fluorescence software (LAS AF 2.6). All images were taken after growth of strains for 2 days on BMM-covered slides as described above. Fluorescence was detected *via* fluorophore excitation using a 488 nm (EGFP) argon laser or 561 nm (mRFP, mCherry) diode-pumped solid-state (DPSS) laser. Images of DAPI-stained hyphae were taken at 405 nm excitation using a 405 diode laser. Recorded images were converted with Metamorph or LasX and further processed with Photoshop CS6 and ImageJ.

## Results

### The Pheromone Response Pathway Regulates Sexual Development, Hyphal Fusion, and Vegetative Growth

Genome sequencing revealed that genes for conserved MAPK signaling pathways exist in *S. macrospora* (Nowrousian et al., 2010). Among these are component homologs of the yeast PR signaling complex (Zhou et al., 1993). In *S. macrospora*, the PR kinase homologs are encoded by *SMAC_03492* (*mak2*), *SMAC_06526* (*mek2*), and *SMAC_05356* (*mik2*).

We further identified the gene for HAM5 (*SMAC_02471*), which is a homolog of a PR pathway scaffold protein in the filamentous fungi *Aspergillus nidulans* and *N. crassa* (Dettmann et al., 2014; Jonkers et al., 2014; Frawley et al., 2018). Deletion strains for *mak2*, *mek2*, *mik2*, and *ham5* were generated using a Δku70 recipient strain for homologous recombination (Pöggeler and Kück, 2006). In general, the target gene is replaced in our deletion strategy by the hygromycin resistance cassette. Thus, deletion strains are easily identified in growth tests. In case of the Δmak2 strain, the hygromycin resistance cassette was removed using the FLP/*FRT* recombination system (Kopke et al., 2010; Teichert et al., 2017). For all strains, ascospore isolates were used for further experiments.

**Figure 1:**
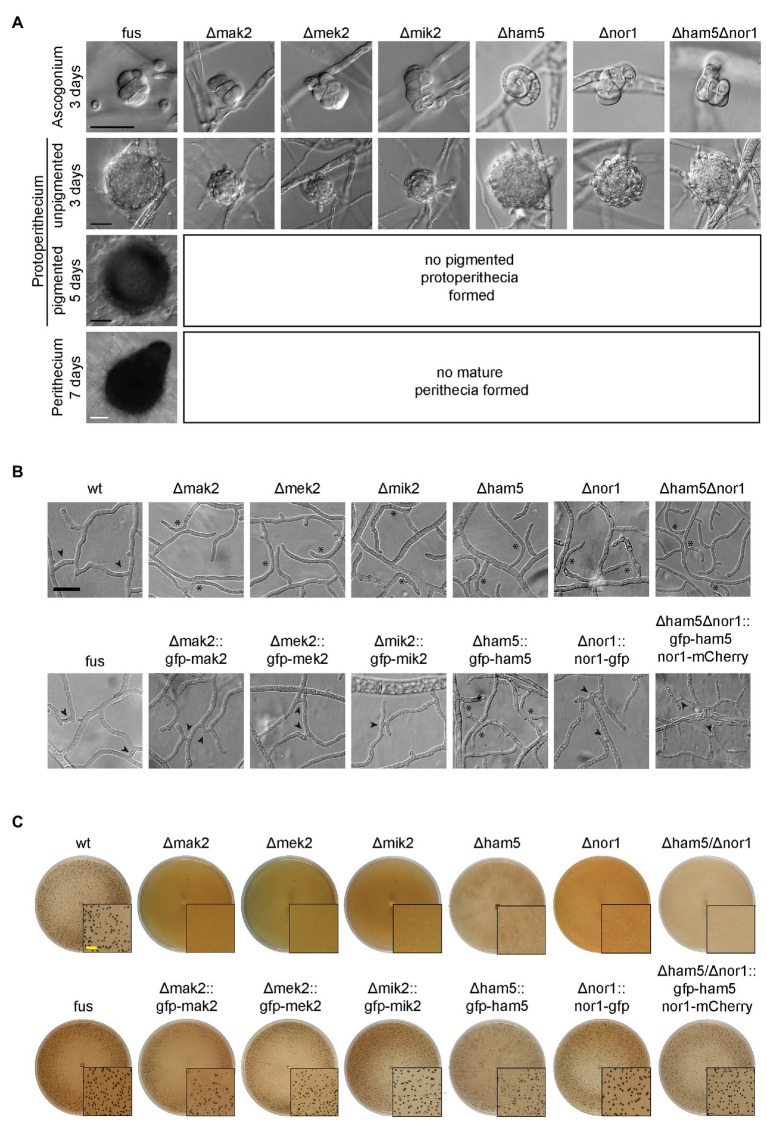
Phenotype of *mak2*, *mek2*, *mik2*, and *ham5* deletion mutants. **(A)** All pheromone response (PR) deletion strains as well as Δnor1 and Δham5Δnor1 show an arrest of fruiting body formation at the stage of unpigmented protoperithecia. Sexual development of deletion strains was observed after 3 (ascogonia), 5 (unpigmented and pigmented protoperithecia) and 7 days (perithecia). All examined strains carry the *fus1-1* mutation. **(B)** Hyphal fusion is impaired in all PR kinase deletion strains as well as in Δnor1 and Δham5Δnor1. Hyphal fusion bridges (arrowheads) were observed in all control (fus and wild type) and complemented strains, but were lacking in the investigated deletion strains (asterisks) after 2 days of growth on cellophane-covered MMS. The hyphal fusion defect was restored in all strains after introduction of the corresponding GFP-fusion constructs, except for Δham5. **(C)** Expression of gfp-tagged fusion constructs of PR genes and *gfp/mCherry*-tagged *nor1* restored fertility in the corresponding deletion strains. All strains were grown on solid BMM for 7 days. Scale bars indicate 20 μm (black), 100 μm (white), or 2 mm (yellow). Developmental phenotypes of Δham5 were identical in wild type and fus mutant strains.

The deletion strains Δmak2, Δmek2, Δmik2, and Δham5 were sterile, while the wild type generated mature perithecia within 7 days (Figure 1A). Similar to the wild type, all deletion strains formed ascogonia and unpigmented protoperithecia after 3 days of incubation. Interestingly, the ascogonia showed wild type like septation, which distinguishes them from sterile *S. macrospora* STRIPAK mutants (Supplementary Figure S6; Radchenko et al., 2018). Furthermore, all strains lacked the ability to form hyphal fusions (Figure 1B). To confirm that the mutant phenotype was caused by the gene deletions, the corresponding wild type genes were fused to *gfp*, and the constructs were transformed into the corresponding deletion strains. In all cases, sexual development and hyphal fusion were restored. One exception was the complemented Δham5 strain, which showed full sexual fertility with viable ascospores, but retained the hyphal fusion defect (Figures 1B,C). This result was identical for N- or C-terminal fusion of HAM5 with GFP (Supplementary Figure S7). HAM5 shares a similar primary structure with its homolog HAM5 from *N. crassa* containing seven N-terminal WD40 domains as well as a C-terminal coiled-coil domain (Supplementary Figure S8).

**Figure 2:**
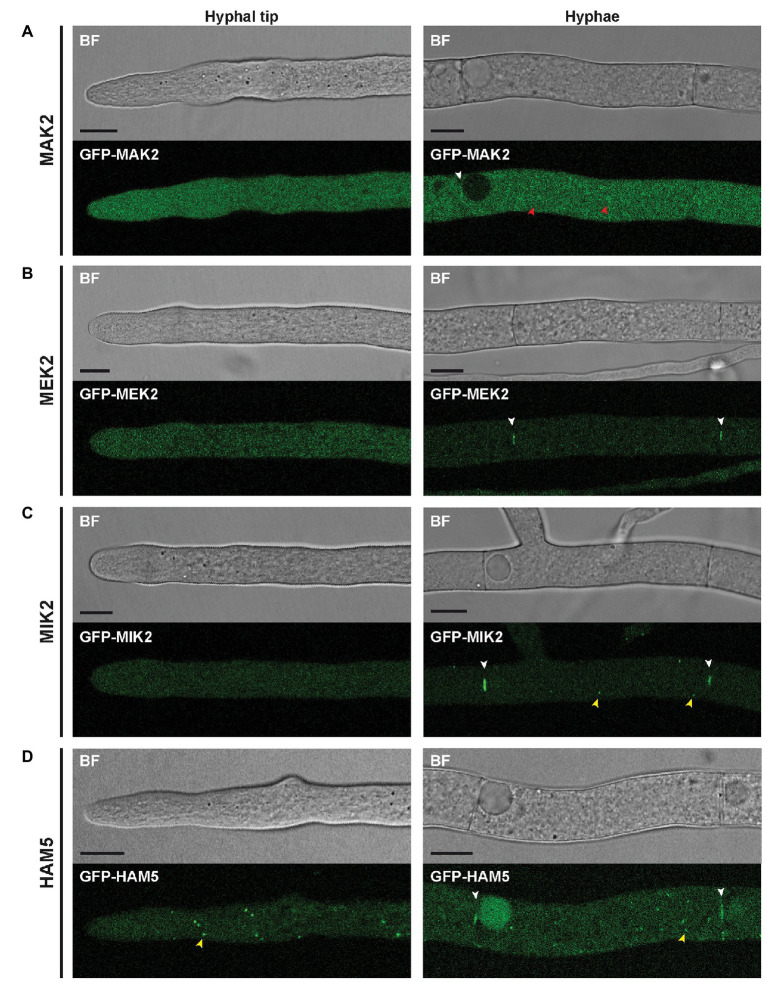
Localization of PR components in hyphae. **(A–D)** All PR components are recruited to septal pores (white arrowheads). **(A)** GFP-MAK2 localizes to the cytoplasm near hyphal tips and additionally shows enhanced fluorescence in spherical structures in mature hyphae (red arrowhead). **(B)** GFP-MEK2 shows faint cytoplasmic localization near hyphal tips and in mature hyphae. **(C)** GFP-MIK2 displays a cytoplasmic distribution near hyphal tips and localizes to small spots (yellow arrowheads) in mature hyphae. **(D)** Compared to MEK2 and MIK2, the more abundant GFP-HAM5 spots are larger in size and can be found close to hyphal tips as well as in mature hyphae (yellow arrowhead). All scale bars indicate 10 μm. BF, bright-field (Confocal microscopy).

**Figure 3:**
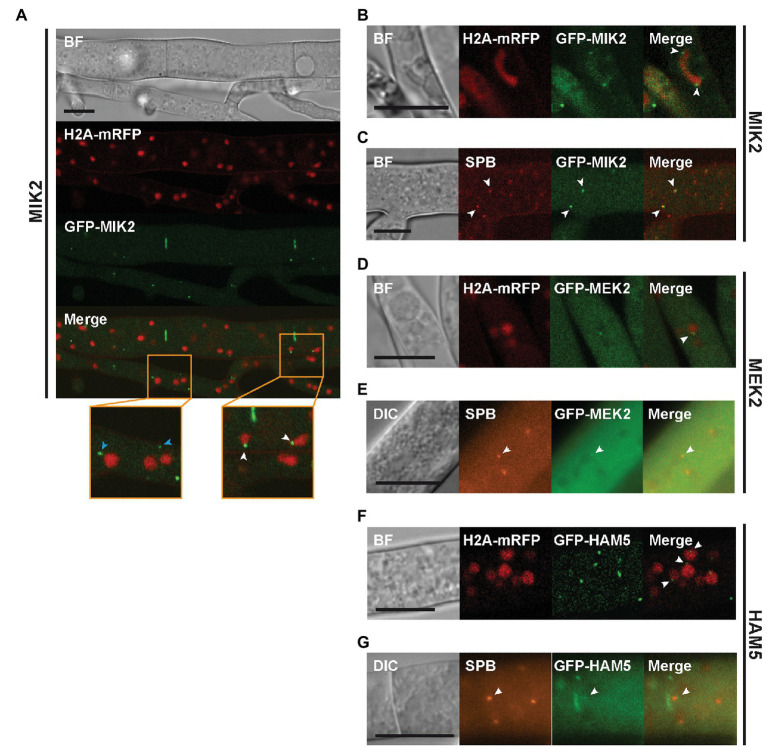
Localization of MIK2 and MEK2 to spindle pole bodies (SPBs). **(A)** Spots of GFP-MIK2 can be observed in the cytoplasm (blue arrowheads) and in association with nuclei (white arrowheads). **(B)** Dividing nuclei show punctate GFP-MIK2 fluorescence at both ends (white arrowheads), suggesting a localization at SPBs. **(C)** Co-expression of GFP-MIK2 with the SPB marker mRFP-GRC1/TUB4-mCherry shows that MIK2 partially accumulates at SPBs (white arrowheads). **(D)** GFP-MEK2 localizes to nuclei associated spots. **(E)** Co-labeling with the SPB marker mRFP-GRC1/TUB4-mCherry confirms MEK2 targeting to SPBs. **(F)** GFP-HAM5 localizes to nuclei-associated spots. **(G)** Co-labeling of GFP-HAM5 with the SPB marker shows partial localization of HAM5 at SPBs. All scale bars represent 10 μm. BF, bright-field (Confocal microscopy); DIC, differential interference contrast (Conventional fluorescence microscopy).

Next, strains expressing *gfp*-fusion genes were used for localization studies of the PR components. Fluorescence microscopy revealed that all PR kinases and HAM5 localize around septal pores (Figure 2). In addition, MAK2 displayed a cytoplasmic localization near the hyphal tip and showed a slight accumulation in spherical structures in mature hyphae (Figure 2A). A different localization was seen for MEK2, MIK2, and HAM5, which localized to small spots (Figures 2C,D, 3D). MEK2 and MIK2 showed a cytoplasmic distribution near the hyphal tip and only exhibited spot-like localization in mature hyphae starting with the third compartment behind the hyphal tip. However, HAM5 spots were distributed throughout the whole length of the hyphae, including the tip compartment. Co-expression of the nuclear marker H2A-mRFP revealed that spots observed for GFP-MIK2, GFP-MEK2, and GFP-HAM5 are sometimes associated with nuclei (Figures 3A,D,F). Furthermore, the localization of two MIK2 spots to both sides of a mitotic nucleus suggests an association with SPBs (Figure 3B).

To confirm the SPB association, we constructed two SPB marker genes, encoding mRFP-GRC1 or TUB4-mCherry. Both GRC1 and TUB4 are core components of the γ-tubulin complex, which is a part of SPBs (Kilmartin, 2014). It has been described for *S. cerevisiae* that overexpression of just one of these genes was lethal, while co-expression of both genes resulted in viable transformants (Knop et al., 1997). Therefore, mRFP-GRC1 and TUB4-mCherry were co-expressed in the wild type strain. We obtained viable ascospores, and the isolates were used for counterstaining with DAPI, demonstrating the expected association of the SPB markers with nuclei (Supplementary Figure S9). Subsequent experiments with the PR kinases revealed partial co-localization of MIK2 and MEK2 spots with SPBs (Figures 3C,E). Similar cellular distribution of PR kinases was reported for *A. nidulans*, where the kinases locate not only to cytoplasmic spots and septa, but also associate with nuclei (Bayram et al., 2012). As a further result, we present the partial co-localization of HAM5 with SPB markers, suggesting that HAM5 is scaffolding PR kinases at the SPBs (Figure 3G).

### Physical and Genetic Interaction Between the PR and NOX Modules

The sterile and hyphal fusion phenotypes observed for the PR deletion mutants are strikingly similar to mutants lacking components of the NADPH oxidase 1 (NOX1) complex. Moreover, ascospore isolates of Δmak2, Δmek2, and Δmik2, but not Δham5, were only recovered when carrying the *fus* genetic background (see below), a phenotype previously observed in NOX2 mutants. Remarkably, mutants lacking NOR1, which is the regulator of the two NOX complexes, exhibit all defects described above (Dirschnabel et al., 2014).

**Figure 4:**
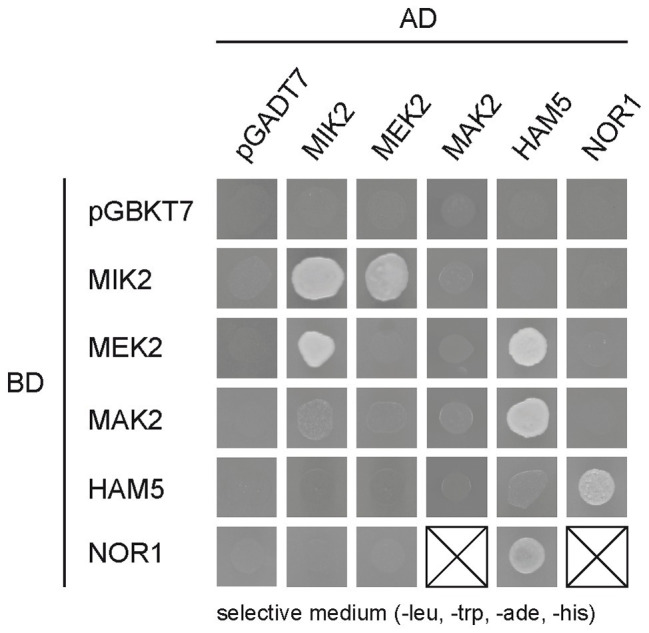
Interaction of NOX regulator (NOR1) with components of the PR pathway. Yeast two-hybrid (Y2H) analysis of PR pathway components and NOX component NOR1. Interaction of fusion proteins was tested by measuring the growth of diploid strains after drop-plating of yeast cells on synthetic defined (SD) medium lacking leucine, tryptophan, adenine, and histidine. The corresponding growth control is shown in supplementary Figure S10 in the supplemental material.

To first address a possible interaction between the PR and NOX modules, we performed a Y2H analysis to test for interactions between components of the PR pathway and the regulator NOR1. First, we constructed plasmids encoding GAL4 activation domain (AD) and GAL4 DNA-binding domain (BD) fusions with MAK2, MEK2, MIK2, HAM5, and NOR1. After mating AD and BD fusion strains, diploid strains were tested for growth (Supplementary Figure S10) and reporter gene activity (Figure 4). Similar to data obtained with *N. crassa* (Dettmann et al., 2014), we found a physical interaction between MIK2 and MEK2, as well as an interaction between HAM5 and both MEK2 and MAK2. In addition, we detected a so far unknown interaction between HAM5 and NOR1, indicating crosstalk between the PR pathway and the NOX complexes (Figure 4).

**Figure 5:**
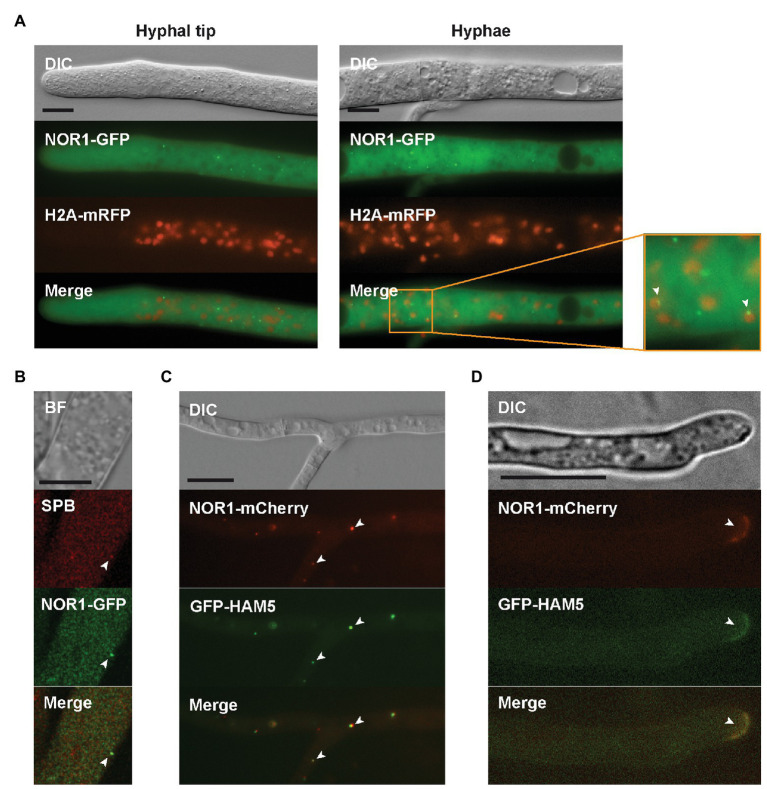
Localization of NOR1. **(A)** NOR1-GFP localizes to the cytoplasm and to cytoplasmic spots in mature hyphae and near the hyphal tip. Co-expression with H2A-mRFP reveals an association of NOR1-GFP spots with nuclei as indicated by white arrowheads. **(B)** Confocal imaging of the Δnor1 strain. NOR1-GFP and the SPB marker show partial co-localization (white arrowheads). **(C)** GFP-HAM5 and NOR1-mCherry co-localize in cytoplasmic spots (white arrowheads) of mature hyphae in the double deletion strain Δham5Δnor1. **(D)** Same as **(C)** but showing co-localization of HAM5 and NOR1 at the plasma membrane of hyphal tips (white arrowhead). Scale bars indicate 10 μm. BF, bright field; DIC, Differential interference contrast.

Next, we used microscopy to further support the interaction between HAM5 and NOR1. Δnor1 was transformed with the *gfp-nor1* construct, and the functionality of NOR1-GFP in Δnor1 was confirmed by restoration of fertility and hyphal fusion in the recombinant strains. Fluorescent imaging of NOR1-GFP revealed a localization pattern similar to that observed for GFP-HAM5, as shown above. This includes a spot-like localization close to the hyphal tip and accumulation around septal pores in mature hyphae. As for MEK2, MIK2, and HAM5, NOR1-GFP spots were found in association with nuclei and showed co-localization with the SPB marker in rare instances (Figures 5A,B).

To examine the HAM5-NOR1 interaction in more detail, the double deletion strain Δham5Δnor1 was constructed by crossing Δham5 with Δnor1. The resulting double mutant was sterile and lacked hyphal fusions (Figure 1). For co-localization studies, genes for GFP-HAM5 and mCherry-NOR1 were co-expressed in the double deletion strain, which resulted in restoration of fertility and hyphal fusion (Figure 1). GFP-HAM5 and NOR1-mCherry were co-localized at plasma membranes of hyphal tips from side branches and further at spots in mature hyphae (Figures 5C,D).

**Figure 6:**
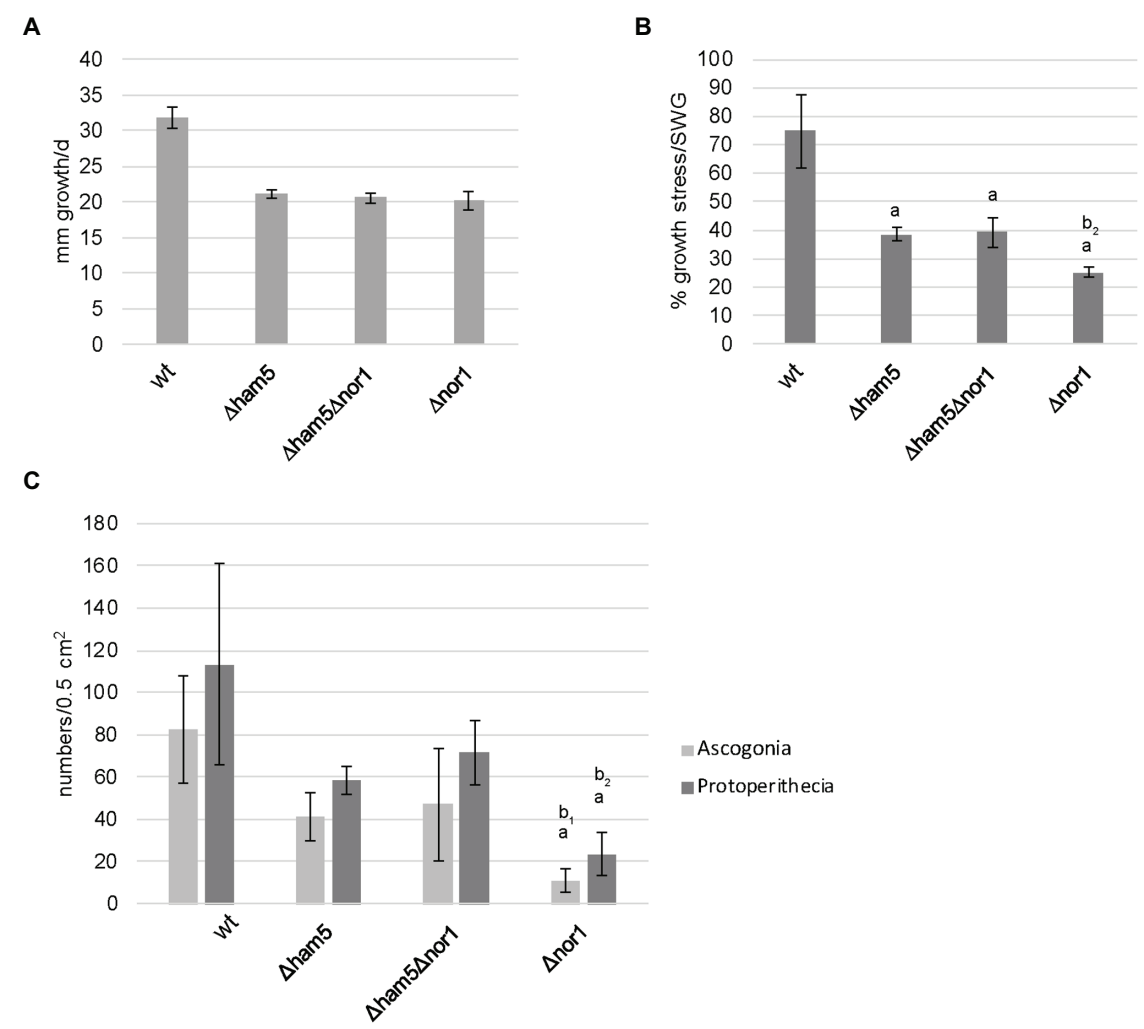
Double deletion of *ham5* and *nor1* suppresses Δnor1 phenotype during the oxidative stress response and the initiation of fruiting body formation. **(A)** Vegetative growth tests were performed with wild type, ∆ham5, ∆ham5∆nor1, and ∆nor1. Strains were grown in Petri dishes on solid synthetic Westergaard’s medium (SWG) at 27°C for 2 days. The growth front was marked after 24 and 48 h. The graph shows means and SDs from three biological replicates per strain. **(B)** Stress-related growth analysis was conducted with strains described in **(A)** on solid SWG medium containing 0.01% H_2_O_2_ at 27°C for 2 days. The growth front was marked after 24 and 48 h. The ratio of the growth rates on SWG and SWG with 0.01% H_2_O_2_ is shown in percentage (%). The graph shows means and SDs from three biological replicates per strain. **(C)** The quantification of ascogonia and protoperithecia of wild type, ∆ham5, ∆ham5∆nor1, and ∆nor1. Graphs represent means and SDs of three biological replicats per strain. For each strain, ascogonia and protoperithecia were counted in an area of 0.5 cm^2^, located 1 cm behind the growth front. Statistical analysis was performed using students *t*-test. Significant differences (*p* ≤ 0.05) to fus (a), Δham5/fus (b_1_) or Δham5/fus and Δham5Δnor1/fus (b_2_) are indicated on top of each bar. All examined strains carry the *fus1-1* mutation.

Next, to test the genetic interaction between NOR1 and HAM5, we conducted vegetative and stress-related growth tests using Δham5, Δnor1, and Δham5Δnor1 mutants (Figures 6A,B). While the growth rate for all the three strains was similar, the response to oxidative stress (H_2_O_2_) was more impaired in Δnor1 than in the two other strains. Δnor1 showed a growth reduction of about 75%, while both the two other strains showed a growth reduction of about 60%. Thus, the double deletion strain Δham5Δnor1 showed the same phenotype as Δham5, indicating that additional deletion of *ham5* can partially suppress the more severe phenotype of the Δnor1 strain. A similar phenotypic overlap was seen when ascogonia and protoperithecia were counted microscopically in an area of 0.5 cm^2^ in wild type and mutant strains. Compared to the wild type, Δham5 and Δham5Δnor1 did not significantly deviate in ascogonia and protoperithecia numbers (Figure 6C). In contrast, a significant reduction in the number of ascogonia and protoperithecia was found for Δnor1, thus supporting our previous assumption that the more severe phenotype of Δnor1 is partially suppressed in the double deletion strain Δham5Δnor1.

### PR Kinases and NOX2 Subunits Are Involved in the Regulation of Ascospore Germination

Interestingly, when we generated ascospore isolates from all PR deletion strains, all the kinase deletion strains turned out to be derived from brown ascospores, i.e., the fus mutant, which is impaired in the biosynthesis of melanin (Engh et al., 2007a). Black, i.e., wild type, ascospore isolates carrying the gene deletions were never recovered. This phenotype is reminiscent of previously characterized mutants, lacking subunits of the NOX2 complex (Dirschnabel et al., 2014). We thus performed a genetic analysis to assess the viability of black and brown ascospores carrying PR deletions.

To quantify the germination rate in black and brown ascospores, Δmak2/fus, Δmek2/fus, Δmik2/fus, and Δham5/fus were crossed against wild type. From each cross, mature recombinant perithecia were used to isolate black and brown ascospores until 100 ascospores of each color had germinated. These isolates were then tested for the PR deletion by growth analysis. Since the deletion strains contain the hygromycin resistance cassette, they are able to grow on antibiotic-containing media. One exception is the Δmak2 strain, which lacks the hygromycin resistance cassette (Supplementary Figure S1). In this case, a sterile phenotype indicates the deletion of the *mak2* gene.

For a gene that does not affect ascospore germination, a 1:1 ratio of deletion and wild type isolates can be predicted for each ascospore color. However, for some of the crosses, 100% wild type black ascospores was recovered (Table 1). This deviation was observed for all kinase deletion strains, and identical results were previously obtained when strains were investigated lacking subunits of the NOX2 complex, including NOR1 (Dirschnabel et al., 2014; Table 1). In contrast, for the Δham5 strain, lacking the scaffold protein of the MAK2 pathway, we obtained a ratio of 1:1. For brown ascospores, all crosses produced wild type and deletion strains in the expected ratio of 1:1. Taken together, these data indicate that the melanin-dependent germination of ascospores is regulated not only by the NOX2 complex but also the HAM5-independent PR pathway.

**Table 1:** Frequency of wild type and mutant phenotypes in black- and brown-spored progeny from indicated crosses.

Cross	Black ascospores		Brown ascospores		Reference
	WT	Δ	WT	Δ	
Δmik2/fus × wt	100%	0%	78%	22%	This work
Δmek2/fus × wt	100%	0%	66%	34%	This work
Δmak2/fus × wt	100%	0%	56%	44%	This work
Δham5/fus × wt	53%	47%	53%	47%	This work
Δnor1/fus × wt	100%	0%	48%	52%	^1^
Δnox1/fus × wt	65%	35%	49%	51%	^1^
Δnox2/fus × wt	100%	0%	43%	57%	^1^

Ascospores were isolated from crosses of the wild type strain against mutant strains carrying the *fus1-1* mutation. 100 (wt × Δham5) or 50 (wt × Δmak2/Δmek2/Δmik2) germinated spores were collected for both spore colors (black and brown). Strains from germinated ascospores were tested for hygromycin resistance or fruiting body formation.

^1^Data from Dirschnabel et al. (2014).

## Discussion

In this study, we show that one of the highly conserved kinase cascades involved in signal transfer during many cellular processes, the PR pathway, is essential for sexual development, hyphal fusion, and ascospore germination in *S. macrospora*. Furthermore, our results indicate a link between the PR pathway and NOX complexes during different cellular processes.

The PR pathway comprises three MAP kinases and the scaffold protein HAM5, which is conserved among filamentous ascomycetes. HAM5 was first described as a scaffold for the PR pathway in *N. crassa*. There, HAM5 was shown to physically interact with either all PR kinases (Jonkers et al., 2014) or only with MAK2 and the kinase STE-50 upstream of the PR pathway (Dettmann et al., 2014). Here, we show that HAM5 interacts with the two downstream kinases MAK2 and MEK2.

Our observations fit well to the described characteristics of scaffolds. These proteins regulate signal transduction pathways by different means, i.e., by regulating the subcellular distribution and the composition of the pathway or by regulating crosstalk and feedback mechanisms within the cascade (Pan et al., 2012). However, signaling cascades are not always regulated by the same scaffold protein. For example, it was reported that the scaffolding activity of the CWI pathway scaffold PRO40 may not be required for all pathway functions (Teichert et al., 2014b). Furthermore, the homologous ERK cascade in humans can be regulated by a variety of scaffolds, which leads to different signaling outputs (Kolch, 2005). Similarly, we propose that HAM5 functions as a scaffold of the PR pathway during hyphal fusion and sexual development, but not during germination of ascospores. Whether the PR kinases operate individually or in association with a different scaffold during this process remains to be investigated.

### Scaffold HAM5 and Regulator NOR1 Connect the PR Pathway and the NOX1 Complex

We found that the scaffold protein HAM5 not only regulates the signal transduction of the PR kinase cascade, but also connects PR pathway with the NOX1 complex. A link between the PR pathway and NOX complexes has often been suggested based on their shared phenotypes (Cano-Domínguez et al., 2008; Serrano et al., 2018). In *S. macrospora*, NOX1 complex deletion strains show similar phenotypes to the PR deletion strains, including defects in sexual development, hyphal fusion, and vegetative growth (Dirschnabel et al., 2014). In *N. crassa*, both complexes are also involved in the formation of aerial hyphae (Cano-Domínguez et al., 2008).

Our Y2H experiments demonstrated that a connection between the PR pathway and the NOX1 complex is mediated *via* a physical interaction between HAM5 and the NOX regulator (NOR1). To further investigate the crosstalk between these pathways, we generated the double deletion strain Δham5Δnor1. Phenotypic analysis revealed that the more severe phenotype of Δnor1 during initiation of sexual development was partially suppressed by the additional deletion of *ham5*. Partial suppression of the Δnor1 phenotype could also be observed during the response to oxidative stress, which has been linked to the PR pathway and the NOX complexes in *N. crassa* and *Botrytis cinerea* (Maerz et al., 2008; Segmüller et al., 2008).

Phenotypic suppression is a phenomenon that can indicate the involvement of two different pathways during regulation of the same process (Prelich, 1999; van Leeuwen et al., 2017). Therefore, we assume that the PR pathway and the NOX1 complex act together during sexual development and the oxidative stress response. Furthermore, both signaling modules may be linked during hyphal fusion, since our analysis indicates that overexpression of *ham5* and *nor1*, but not of *ham5* alone, can restore hyphal fusion in the corresponding mutants. We hypothesize that the HAM5-NOR1-mediated interaction of PR and NOX1 complex signaling is necessary for the regulation of hyphal fusion. This PR and NOX1 signaling can be disrupted by an increased synthesis of HAM5 in the complemented strains, since scaffold concentrations that are higher than the optimum will lead to an incomplete assembly of the cascade (Levchenko et al., 2000; Ortiz-Muñoz et al., 2020). This would increase the likelihood that NOR1 associates with an incompletely assembled PR complex, and thus leads to inhibition of signal transduction. High concentrations of both, HAM5 and NOR1, like in the double deletion strain complemented with both genes, increase the possibility that NOR1 interacts with a fully assembled PR complex. How the two pathways interact with each other during this process remains to be investigated.

Germling fusion, a process similar to fusion of mature hyphae, is well investigated in *N. crassa*. Both the NOX-1 complex and PR kinases are required for this process (Fischer and Glass, 2019). Deletion of *nor1* and *nox1* homologs does not affect the phosphorylation status of the MAK2 homolog during germling fusion (Serrano et al., 2018). It was shown that NOX-1 regulates the transcription of genes responsible for cell communication and cell fusion; however, it remains unknown how this response is triggered (Dirschnabel et al., 2014; Fischer and Glass, 2019). The mammalian homolog of NOR1, p67^phox^, can be phosphorylated by the MAPK ERK2 *in vitro* as well as in stimulated neutrophils (Dang et al., 2003), suggesting that MAPK signaling operates upstream of NOX signaling. However, NOX complex components were not identified as phosphorylation targets of MAK2 in *N. crassa* (Jonkers et al., 2014).

To better understand how NOR1 and PR components interact, we investigated their subcellular distribution. We observed a localization of PR components to cytoplasmic spots and septa, similar to reports for *N. crassa* and *A. nidulans* (Bayram et al., 2012; Jonkers et al., 2014; Frawley et al., 2018). Furthermore, time-lapse imaging in *A. nidulans* revealed that spot-like accumulations of the PR complex migrate between the plasma membrane and the nuclear envelope (Bayram et al., 2012). We found a similar spot-like localization for NOR1, which is reminiscent of observations in *Epichloë festucae* (Takemoto et al., 2011). It has been hypothesized that septal pores act as signaling hubs in filamentous fungi, since proteins of many different pathways have been associated with this compartment, including components of the CWI pathway, the septation initiation network (SIN), and the morphogenesis Orb6 (MOR) network (Seiler et al., 2006; Seiler and Justa-Schuch, 2010; Riquelme et al., 2011; Dettmann et al., 2012; Heilig et al., 2013, 2014). In *N. crassa*, the association of the PR pathway with the septal pore might be independent of the scaffolding function of HAM5 (Jonkers et al., 2014).

We also observed that PR components and NOR1 localize to SPBs. To our knowledge, these components have not yet been found at SPBs in filamentous fungi. Similar to septal pores, SPBs have been suggested to act as signaling hubs for the regulation of cell cycle progression (Pereira and Schiebel, 2001). A link between cell cycle regulation and the PR pathway was observed in yeast, where triggering of the PR leads to cell cycle arrest in the G1-phase and initiation of growth toward the mating partner (Hilioti et al., 2008). Furthermore, homologs of the PR pathway and NOX complexes in mammals have been associated with functions of the centrosome (Colello et al., 2012; Kalive and Capco, 2013; Arnandis et al., 2018). However, whether these pathways interact at these two signaling sites is still an open question.

Interestingly, HAM5 and NOR1 co-localize to hyphal tips of side branches as well as to spots in mature hyphae. This co-localization of PR and NOX components hints at a connection between these pathways during hyphal fusion or sexual development, since both these processes occur in the older part of the mycelium. Indeed, co-localization in cytoplasmic spots was also observed for NOR-1 and NOX-1 during germling fusion in *N. crassa* and *E. festucae*. However, this co-localization is not observed in other developmental stages. NOX-1 was described to localize to the endoplasmic reticulum and the vacuolar system, while NOR-1 was found at cytoplasmic spots (Siegmund et al., 2013; Cano-Domínguez et al., 2019).

### HAM5 Is Not Involved in the Link Between PR and NOX2 During Ascospore Germination

PR kinase deletion strains not only share similar phenotypes with the NOX1, but also with the NOX2 complex. Both PR and NOX2 are essential for melanin-dependent ascospore germination. Previously, it was proposed that the production of ROS by the NOX complex is required for ascospore germination. If NOX2 complex components were deleted, the residual ROS from other sources would be scavenged by melanin in the cell wall, and would thus not be sufficient to induce germination. However, in melanin-deficient strains, smaller amounts of ROS would be sufficient to initiate this process (Dirschnabel et al., 2014).

HAM5 is apparently not involved in linking PR and NOX2 during ascospore germination, since we found that the *ham5* deletion strain does not have a melanin-dependent ascospore germination defect. These findings are similar to results obtained with *P. anserina* (Malagnac et al., 2004; Jamet-Vierny et al., 2007; Lambou et al., 2008; Lalucque et al., 2012). It remains to be investigated whether the PR kinases are regulated by a different scaffold during this process, and if there is a direct connection to the NOX2 complex. Regulation by several scaffolds can be seen for the homologous extracellular signal-regulated kinase (ERK) pathway in humans. Scaffold proteins participate in the regulation of spatio-temporal signaling by targeting the ERK pathway to specific cellular compartments, such as the plasma membrane, Golgi apparatus, or endosomes (Wortzel and Seger, 2011). Furthermore, scaffold proteins can also influence the composition of the cascade, which can be seen for the scaffold protein Ste5, which is required for correct assembly of the PR pathway in *S. cerevisiae*. In the absence of Ste5, the downstream kinase of the cascade switches from Fus3 to Kss1, and thus changes the signaling output (Witzel et al., 2012).

In conclusion, we discovered that the *S. macrospora* PR pathway is involved in several developmental processes, including sexual development and hyphal fusion. Our interaction studies indicate that physical interaction between the PR scaffold HAM5 and the NOX regulator (NOR1) mediates the crosstalk between the PR pathway and the NOX1 complex during sexual development, hyphal fusion, and oxidative stress response. In contrast, crosstalk between PR and NOX2 signaling does not require HAM5 during melanin-dependent ascospore germination.

## Data Availability Statement

The raw data supporting the conclusion of this article will be made available by the authors, without undue reservation, to any qualified researcher.

## Author Contributions

SS, RM, BR, and AB-R performed the experiments. SS acquired microscopic images. RM and AB-R generated mutant strains. IT and UK conceived this study and supervised the experiments. SS, IT, and UK wrote the manuscript and designed the figures. All authors contributed to the article and approved the submitted version.

### Conflict of Interest

The authors declare that the research was conducted in the absence of any commercial or financial relationships that could be construed as a potential conflict of interest.
