# Supplementary material for: Crosstalk Between Pheromone Signaling and NADPH Oxidase Complexes Coordinates Fungal Developmental Processes
Source: Front Microbiol. 2020 Jul 28;11:1722. doi: 10.3389/fmicb.2020.01722 (PMC7401384; doi:10.3389/fmicb.2020.01722)
Supplement: Supplementary file 1 [file Data_Sheet_1.docx]

**Supplements**

Table S1

Table S2

Table S3

**Table S1: Strains used in this study**.

| Strain | **Relevant genotype and phenotype** | **Source / Reference** |
| --- | --- | --- |
| R19027 | Wild type, fertile | Culture collection, AMB^1^ |
| S70823 | Spore color mutant, fertile  *fus1-1* | Culture collection, AMB^1^ |
| DD574 | ASI, sterile  *Δnor1::hph, fus1-1* | Dirschnabel et al (2014) |
| RM1941 | ASI, ectopic integration of pGG-N-EGFP-HAM5 into RM329, fertile  *Δham5::hph, gpd(p)::egfp::ham5::trpC(t), trpC(p)::nat* | This work |
| S143932, S1143995 | ASI, sterile  *Δmek2::hph, fus1-1* | This work |
| S147281, RM329, RM349, S147293, S147294, S147296, S147308 | ASI, sterile  *Δham5::hph, fus1-1* | This work |
| S156605, S156553, S156561 | ASI, sterile  *Δham5::hph, Δnor1::hph, fus1-1* | This work |
| S96888 | Strain for homologous recombination, fertile  *Δku70*::*nat* | Culture collection, AMB^1^ |
| SaS1090, SaS1091, SaS1092, SaS1093 | ASI, sterile  *Δmik2::hph, fus1-1* | This work |
| SaS1097 | ASI, ectopic integration of pH2A-mRFP into R19027, fertile  *gpd(p)::h2a::mrfp1::trpC(t), trpC(p)::nat* | This work |
| SaS1298, S144175, S144315, SaS1298 | ASI, sterile  *Δmak2, fus1-1* | This work |
| SaS1339 | ASI, ectopic integration of pgfp-mik2 into SaS1090, fertile  *∆mik2::hph, gpd(p)::egfp::mik2::trpC(t), trpC(p)::nat, fus1-1* | This work |
| SaS1669 | ASI, ectopic integration of pgfp-mak2 into SaS1298, fertile  *∆mak2, gpd(p)::egfp::mak2::trpC(t), trpC(p)::nat, fus1-1* | This work |
| SaS1787 | ASI, ectopic integration of pgfp-mek2 into S143932, fertile  *∆mek2::hph, gpd(p)::egfp::mek2::trpC(t), trpC(p)::nat, fus1-1* | This work |
| SaS1821 | ASI*,* ectopic integration of pnor1-gfp into DD574, fertile  *∆nor1::hph, gpd(p)::nor1::egfp::trpC(t), trpC(p)::nat, fus1-1* | This work |
| SaS1938 | ASI from crossing of SaS1821 and SaS1097, fertile  *Δnor1::hph, gpd(p)::nor1::egfp::trpC(t), trpC(p)::nat, gpd(p)::h2a::mrfp::trpC(t), trpC(p)::nat, fus1-1* | This work |
| SaS2308 | ASI from crossing of RM1941 and SaS1097, fertile  *Δham5::hph, gpd(p)::egfp::ham5::trpC(t), trpC(p)::nat, gpd(p)::h2a::mrfp::trpC(t), trpC(p)::nat* | This work |
| SaS2372 | ASI, ectopic integration of pGG-mRFP-GRC1 and pTub4-mCherry into R19027, fertile  *gpd(p)::mrfp::grc-1::trpC(t), trpC(p)::nat, gpd(p)::tub4::mcherry::trpC(t), trpC(p)::hph* | This work |
| SaS2451 | ASI from crossing of SaS2372 and S70823, fertile  *gpd(p)::mrfp::grc-1::trpC(t), trpC(p)::nat, gpd(p)::tub4::mcherry::trpC(t), trpC(p)::hph, fus1-1* | This work |
| SaS2538 | ASI from crossing of SaS1821 and SaS2451, fertile  *Δnor1::hph, gpd(p)::nor1::egfp::trpC(t), trpC(p)::nat, gpd(p)::mrfp::grc-1::trpC(t), trpC(p)::nat, gpd(p)::tub4::mcherry::trpC(t), trpC(p)::hph, fus1-1* | This work |
| TSA149A3 | Primary transformant, ectopic integration of pGG-N-EGFP-HAM5 and pNor1-mCherry into S156605*,* fertile  *Δham5::hph, Δnor1::hph, gpd(p)::nor1::mcherry::trpC(t), trpC(p)::nat, gpd(p)::egfp::ham5::trpC(t), trpC(p)::nat, fus1-1* | This work |
| TSA153D3 | Primary transformant, ectopic integration of ptub4-mcherry and pGG-N-EGFP-HAM5 into R19027, fertile  *gpd(p)::tub4::mcherry::trpC(t), trpC(p)::hph, gpd(p)::egfp::ham5::trpC(t), trpC(p)::nat* | This work |

^1^AMB, Allgemeine & Molekulare Botanik, Ruhr-University, Bochum, Germany

**Table S2: Plasmids used in this study**.

| Plasmid | **Relevant features** | **Reference** |
| --- | --- | --- |
| pDS23 | Cloning vector *gpd(p)::egfp::trpC(t)* in pRSnat, *URA3, bla, nat* | Schindler & Nowrousian (2014) |
| pH2A-mRFP | *gpd(p)::h2a::mRFP::trpc(t)*, *URA3, bla, nat* | This work |
| pGG-N-mRFP | *Plasmid for GoldenGate cloning,*  *gpd(p)::mRFP::trpC(t), bla, nat* | Teichert  (unpublished data) |
| pGG-mRFP-GRC1 | *gpd(p)::grc1::mrfp::trpC(t)* in pGG-N-mRFP1,  *bla, nat* | This work |
| pCherry | *gpd(p)::mcherry::trpC(t), bla, hph* | Engh et al. (2010) |
| pTUB4-mCherry | *gpd(p)::tub4::mcherry::trpC(t)* in pCherry, bla*, hph* | This work |
| pEGFP-mik2 | *gpd(p)::egfp::mik2::trpC(t)* in pGG-N-EGFP, *bla, nat* | This work |
| pEGFP-mek2 | *gpd(p)::egfp::mek2::trpC(t)* in pDS23, *URA3, bla, nat* | This work |
| pEGFP-mak2 | *gpd(p)::egfp::mak2::trpC(t)* in pDS23, *URA3, bla, nat* | This work |
| pGG-N-EGFP-HAM5 | *gpd(p)::egfp::ham5::trpC(t)* in pGG-N-EGFP,  *bla, nat* | This work |
| pGG-C-EGFP-HAM5 | *gpd(p)::ham5::egfp::trpC(t)* in pGG-C-EGFP,  *bla, nat* | This work |
| pGG-C-EGFP | *Plasmid for GoldenGate cloning,*  *gpd(p)::egfp::trpC(t), bla, nat* | Teichert  (unpublished data) |
| pGG-N-EGFP | *Plasmid for GoldenGate cloning,*  *gpd(p)::egfp::trpC(t), bla, nat* | Teichert (unpublished data) |
| pGG-Nor1-gfp | *gpd(p)::nor1::egfp::trpC(t)* in pGG-C-EGFP,  *bla, nat* | This work |
| pNor1-mCherry | *gpd(p)::nor1::mcherry::trpC(t)* in pGG-Nor1-gfp,  *bla, nat* | This work |
| pFlip5-MAK2 | Deletion plasmid for SMAC_03492  *smxyl(p)::Pcflp, trpC(p)::hph* | This work |
| pFlip3-MAK2 | Deletion plasmid for SMAC_03492  *smxyl(p)::Pcflp, trpC(p)::hph* | This work |
| pKO-mek2 | Deletion plasmid for SMAC_06526  5’ flank *mek2, trpC(p)::hph,*  3’ flank *mek2, bla, kan^r^* | This work |
| pKO-mik2 | Deletion plasmid for SMAC_05356  5’ flank *mik2, trpC(p)::hph,*  3’ flank *mik2, ura3, bla* | This work |
| pKO-2471 | Deletion plasmid for SMAC_02471  (*ham5*), 5’ flank *ham5*,  trpC(p)::hph, 3’ flank *ham5*,  ura3, bla | This work |
| pKO-nor1 | Deletion plasmid for SMAC_02124, 5’ flank *nor1*,  trpC(p)::hph, 3’ flank *nor1*,  ura3, bla | Dirschnabel et al. (2014) |
| pAmik2 | *mik2* cDNA in pGADT7, *LEU2*, *amp^r^* | This work |
| pBmik2 | *mik2* cDNA in pGBKT7, *TRP1*, *kan^r^* | This work |
| pAmek2 | *mek2* cDNA in pGADT7, *LEU2*, *amp^r^* | This work |
| pBmek2 | *mek2* cDNA in pGBKT7, *TRP1*, *kan^r^* | This work |
| pAmak2 | *mak2* cDNA in pGADT7, *LEU2*, *amp^r^* | This work |
| pBmak2 | *mak2* cDNA in pGBKT7, *TRP1*, *kan^r^* | This work |
| pGBDT7 | Yeast two-hybrid prey plasmid, *S. cerevisiae* *PADH1* and *TADH1*, *gal4-AD* (768-881 aa), *LEU2*, *amp^r^* | Clonetech |
| pGADT7 | Yeast two-hybrid bait plasmid, *S. cerevisiae* *PADH1* and *TADH1*, *gal4-BD* (1-147 aa), *TRP1*, *kan^r^* | Clonetech |
| pAham5 | *ham5* cDNA in pGADT7, *LEU2*, *amp^r^* | This work |
| pBham5 | *ham5* cDNA in pGBKT7, *TRP1*, *kan^r^* | This work |
| pAnor1 | *nor1* cDNA in pGADT7, *LEU2*, *amp^r^* | This work |
| pBnor1 | *nor1* cDNA in pGBKT7, *TRP1*, *kan^r^* | This work |
| pJet1.2blunt | letal gene *eco47IR* for selection of transformants, *amp^r^* | Thermo Fisher Scientific |
| pSF27-34 | *trpC(p)::hph in pDrive, kan^r^, bla* | Nowrousian & Cebula (2005) |
| pMSHnat | *gpd(p)::mrfp::trpC(t), bla, nat* | (Teichert, unpublished data) |

**Table S3: Oligonucleotides used in this study**.

| Oligonucleotide | **Sequence (5’-3’)** | **Specificity** |
| --- | --- | --- |
| 5356_5fw | gtaacgccagggttttcccagtcacgacggaattcggggttttccatggtcgcttgcgtg | *mik2* 5‘ flank forward |
| 5356_5rv | cgagggcaaaggaatagggttccgttgaggggttg  aagcgacggtcagctaggac | *mik2* 5‘ flank reverse |
| 5356_3fw | gcccaaaaatgctccttcaatatcagttgctgatcgttcaatatcatgaggggga | *mik2* 3‘ flank forward |
| 5356_3rv | gcggataacaatttcacacaggaaacagcgaattcgggagataagtcccatgtgtcaaga | *mik2* 3‘ flank reverse |
| KO-1 | gcaggagctcctcctccagttttc | upstream of *mik2* 5‘ flank forward |
| KO-2 | ggaaagcgccgatatttaaagcaatc | downstream of *mik2* 3‘ flank reverse |
| KO-3 | acgactgggaacccaacgagg | *mik2* reverse |
| mek2_5_fw | atcggatccagaattcaacttttgagaagtgacagaa | *mek2* 5‘ flank forward |
| mek2_5_rv | ccgggaaccagttgaggtgtaggtttaagcgtaat | *mek2* 5‘ flank reverse |
| mek2_3_fw | ctacgactggctctcagc | *mek2* 3‘ flank forward |
| mek2_3_rv | gcttgtcgacgaattcggtaagggggtgacgcgg | *mek2* 3‘ flank reverse |
| ptrpC-mek2 | gagagccagtcgtagactgatattgaaggagcattt | *PtrpC* forward |
| hph_rev | tcaactggttcccggtcg | *hph* reverse |
| MEK2KO1 | ctggttgtctccgttcccaaaatc | upstream of *mek2* 5‘ flank forward |
| MEK2KO2 | ctggtggatcatgaacatacg | downstream of *mek2* 3‘ flank reverse |
| hph1MN | cgatggctgtgtagaagtactcgc | *hph* reverse |
| hph2MN | atccgcctggacgactaaaccaa | *hph* forward |
| MAK2-KO1-KpnI | ggtacccacaatattgccctcgaaacg | *mak2* 5‘ flank forward |
| MAK2-KO2-SnaBI | tacgtatttggcgtgtccctgaggg | *mak2* 5‘ flank reverse |
| MAK2-KO3-HindIII | aagcttacgtctacttgcatacaagctgtggg | *mak2* 3‘ flank forward |
| MAK2-KO4-BglII | agatctgtcttgccttgccttgctgg | *mak2* 3‘ flank reverse |
| hph_split_3’_fw | ttggcgacctcgtattgggaatc | *hph* forward |
| hph_split_5’_rv | cgttgcaagacctgcctgaaacc | *hph* split reverse |
| MAK2KO1 | ctcctgtttattcctccatcagct | upstream of *mak2* 5‘ flank forward |
| MAK2KO2 | cctcctcgagagcgaacacatcat | downstream of *mak2* 3‘ flank reverse |
| 2471_5fw_neu | gtaacgccagggttttcccagtcacgacggaattcctcatgacaatgcactgtccagtgg | *ham5* 5‘ flank forward |
| 2471_5rv_neu | cgagggcaaaggaatagggttccgttgaggttgttagtcgggaagctgcatgggc | *ham5* 5‘ flank reverse |
| 2471_3fw | gcccaaaaatgctccttcaatatcagttgcgacgggattgcatcatagtgagatg | *ham5* 3‘ flank forward |
| 2471_3rv | gcggataacaatttcacacaggaaacagcgaattctttgcatttttctgccttgggggtt | *ham5* 3‘ flank reverse |
| 2471_vp1 | aacgagacggacacgcctag | upstream of *ham5* 5‘ flank forward |
| 2471_vp2 | acgtggatgtttcgttgtgtattc | downstream of *ham5* 3‘ flank reverse |
| 2124_fw | ggatccttatgtcgctaaaacaggaaatag | *nor1* forward |
| 2124_rv | ggatccctatatctcctggacccagacc | *nor1* reverse |
| 02124_vp1 | ggacaatttccgaggagctggac | upstream of *nor1* 5‘ flank forward |
| 02124_vp2 | gcttcatgtcagatcgcttgttcc | downstream of *nor1* 3‘ flank reverse |
| GG_01693_for1 | tcactcggtctcgtggtatgggatcatcagcaacc | *grc1* forward |
| GG_01693_rev1 | tcactcggtctcgtacgcgtcatgaccccccacca | *grc1* reverse, *bsaI* mutation |
| GG_01693_for2 | tcactcggtctcggttcctcgtaggggttgta | *grc1* forward, *bsaI* mutation |
| GG_01693_rev2 | tcactcggtctcggaacgcgaccgcttgtcaggg | *grc1* reverse |
| NotI-tub4-for | gctcagcggccgcatgcccaggtacgcgtcc | *tub4* forward |
| EcoRI-tub4-rev | cctggaattcacaagccatccgtttgtccgtg | *tub4* reverse |
| 1757 | agctgacatcgacaccaacg | *trpC(t)* reverse |
| GG-nor1-rev | tctctcggtctccatcccgatatctcctggacccag | *nor1* reverse |
| GG-nor1-for | tctctcggtctcctggtatgtcgctaaaacaggtca | *nor1* forward |
| EcoRV-mCherry-for | gggtccaggagatatcgggatctggctctggtatgg | *mcherry* forward |
| mik2.1_FWD | agagcgtggtctcgtggtatggccatgctggcatcc | *mik2* forward |
| mik2.1_REV | agagcgtggtctcgcagcccaccgaactgacgga | *mik2* reverse |
| mik2.2_FWD | agagcgtggtctcggctgcgaccgcctagcgagc | *mik2* forward, *bsa*I mutation |
| mik2.2_REV | agagcgtggtctcgagatagaccctggaggatctggcg | *mik2* reverse, *bsa*I mutation |
| mik2.3_FWD | agagcgtggtctccatcttacctccacaaccgtg | *mik2* forward, *bsa*I mutation |
| mik2.3_REV | agagcgtggtctcctacgtcatgtccctggcgcagg | *mik2* reverse |
| gfp_mek2_fw | tctcggcatggacgagctgtacaagatgaagcgcaagaacgtcaaaggtc | *mek2* forward |
| gfp_mek2_rv | tgatttcagtaacgttaagtggatccttaaaatcggccggttggaggctgct | *mek2* forward |
| gfp_mak2_ fw | tctcggcatggacgagctgtacaagatgagcagcgcacaaagaggcggag | *mak2* forward |
| gfp_mak2 _rv | tgatttcagtaacgttaagtggatcctcacctcatgatctcgttgtagatca | *mak2* forward |
| ham5-fw1 | tttggtctcctggtatgtcggtccccggacac | *ham5* forward |
| ham5-rv1 | tgtggtctcagagccattccttctttgtctgc | *ham5* reverse |
| ham5-fw2 | tttggtctcagttctcgcagccgcatcc | *ham5* forward |
| ham5-rv3 | tttggtctcgatccgatcatctcactatgatgcaatcc | *ham5* reverse |
| ham5-rv4 | ggtctcttacgtcagatcatctcactatgatgcaatcc | *ham5* reverse |
| 3492-01_AD | atatggccatggaggccagtgaattcatgagcagcgcacaaagagg | *mak2* forward |
| 3492-02_AD | atctgcagctcgagctcgatggatcctcacctcatgatctcgttg | *mak2* forward |
| 3492-01_BD | tgcatatggccatggaggccgaattcatgagcagcgcacaaagagg | *mak2* forward |
| 3492-02_BD | tgcggccgctgcaggtcgacggatcctcacctcatgatctcgttg | *mak2* forward |
| 6526-01_Nde | catatgatggccgacccattcgcc | *mek2* forward |
| 6526-02_Bam | ggatccttaaaatcggccggttggag | *mek2* forward |
| 5356-01_Eco | gaattcatggccatgctggcatcc | *mik2* forward |
| 5356-02_Bam | ggatcctcatgtccctggcgcag | *mik2* reverse |
| 5356-03 | ctctcccatgtcctaccagg | *mik2* forward |
| 5356-04 | tgatctccggttaggctcgg | *mik2* reverse |
| 2471_01AD | acgacgtaccagattacgctcatatgtcggtccccggacacatctcg | *ham5* forward |
| 2471_02 | ttgaggtagggcttgttggacg | *ham5* reverse |
| 2471_03 | acatgtcgcccttagctagg | *ham5* forward |
| 2471_06_BD1 | cagctcgagctcgatggatcctcagatcatctcactatgatg | *ham5* reverse |
