# Supplementary material for: Crosstalk Between Pheromone Signaling and NADPH Oxidase Complexes Coordinates Fungal Developmental Processes
Source: Front Microbiol. 2020 Jul 28;11:1722. doi: 10.3389/fmicb.2020.01722 (PMC7401384; doi:10.3389/fmicb.2020.01722)
Supplement: Supplementary file 2 [file Data_Sheet_2.docx]

**Supplements**

Figure S1

Figure S2

Figure S3

Figure S4

Figure S5

Figure S6

Figure S7

Figure S8

Figure S9

Figure S10


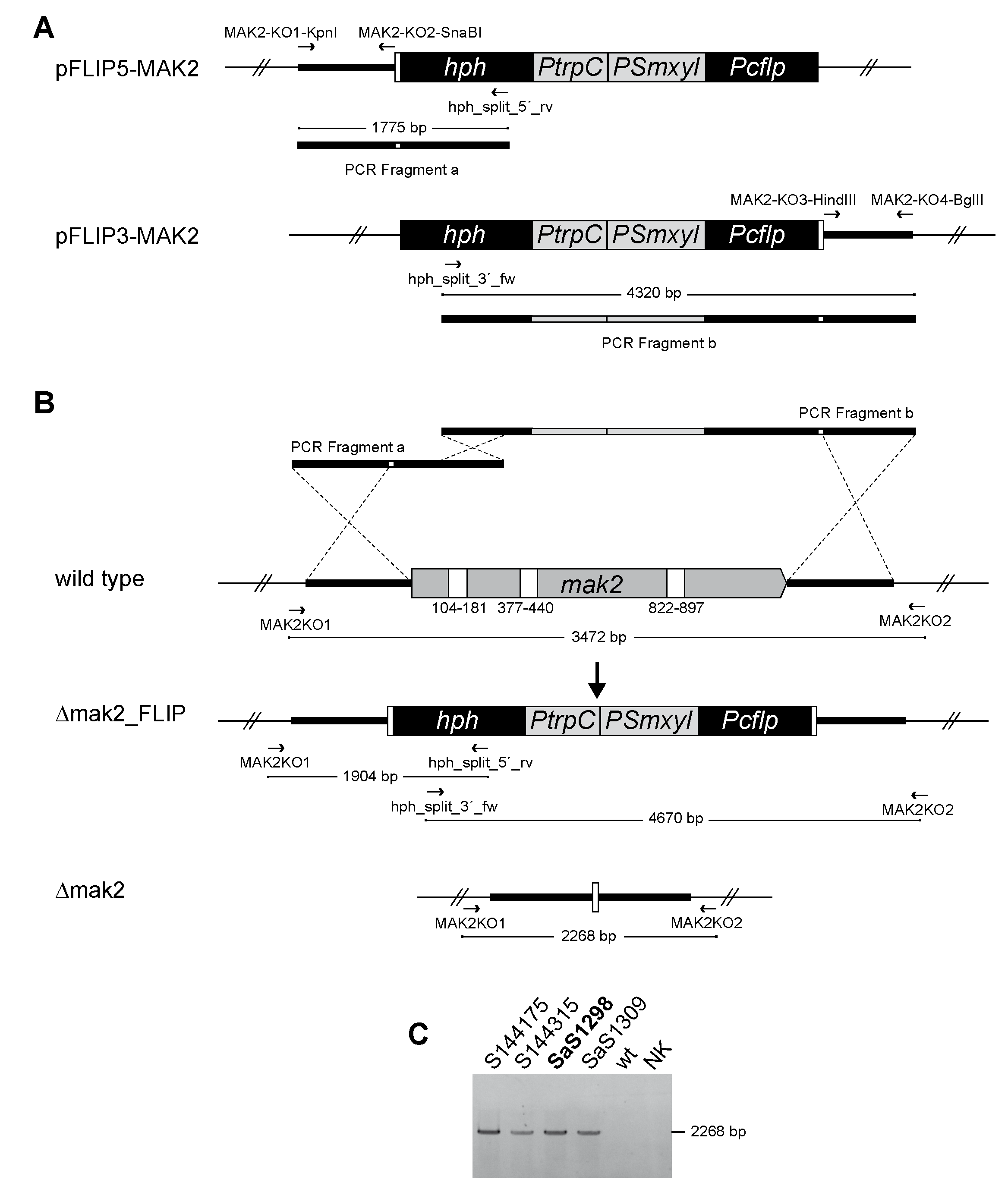


**Figure S1:** Generation and verification of Δmak2 deletion strains. (A) Constructs pFLIP5-MAK2 and pFLIP3-MAK2 carry the cassette for *mak2* deletion using the FRT-FLP recombinant system. Arrows mark binding sites of oligonucleotides used for construction of the deletion plasmid and for verification of the deletion. PCR fragments are indicated with black lines. (B) Genomic situation of the *mak2* locus in wild type (wt), in the Δmak2_FLIP strain after homologous recombination and in Δmak2 after FRT-mediated removal of the deletion cassette. Introns of the *mak2* gene as well as FRT sites in Δmak2_FLIP and Δmak2 are indicated by white bars. Flanking regions are shown as thin black bars and homologous integration is indicated by dotted lines. (C) PCR analysis for verification of the *mak2* deletion strains S144175, S144315, SaS1298, and SaS1309. Deletion of the gene and absence of the deletion cassette was verified using primers MAK2KO1 and MAK2KO2. Wild type (wt) served as a control, where full-length *mak2* was amplified. The negative control (NK) contained no genomic DNA. bp: base pairs.


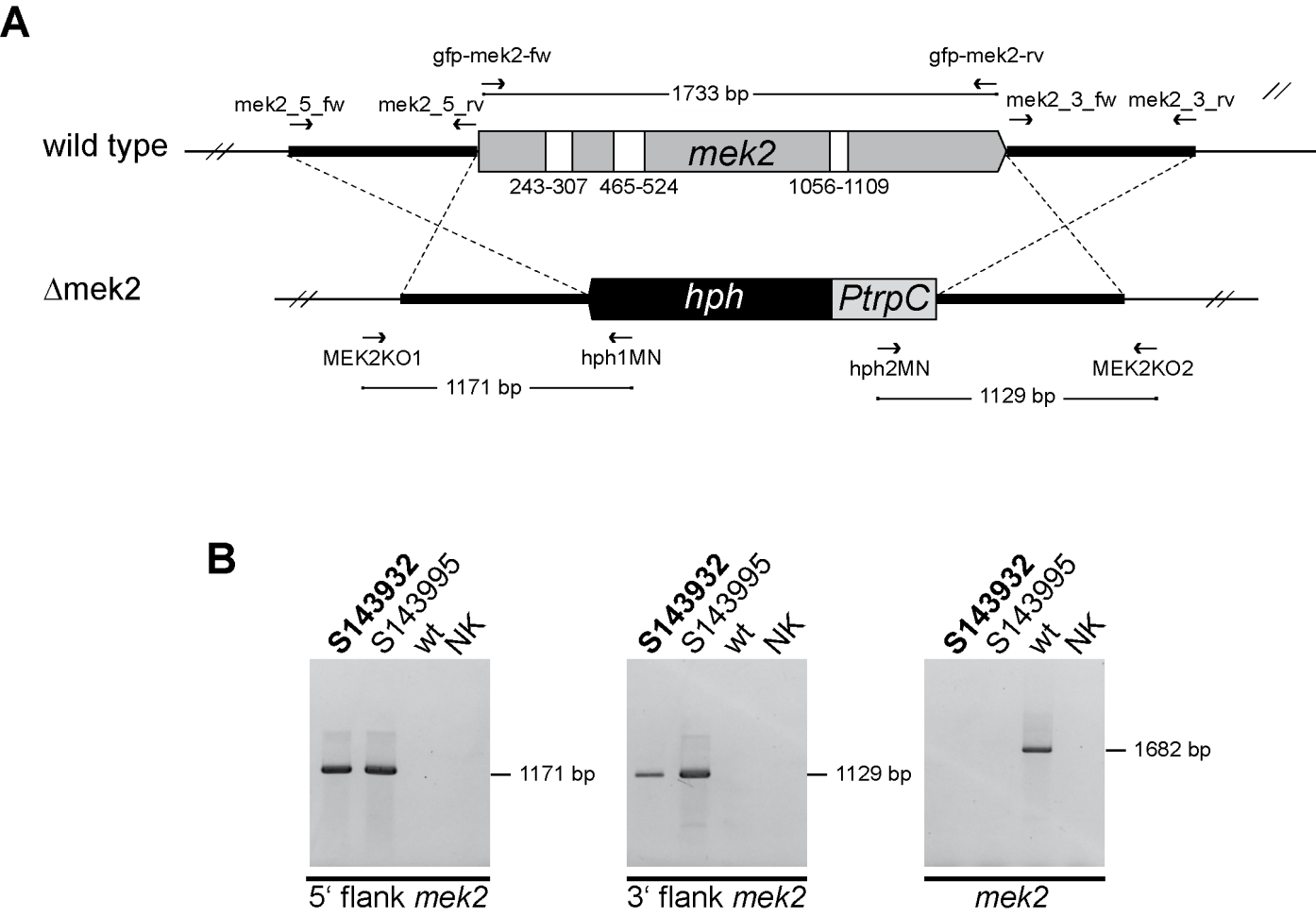


**Figure S2:** Generation and verification of Δmek2 deletion strains. (A) Genomic situation of the *mek2* locus in wild type (wt) and in the Δmek2 strain after homologous recombination. Introns are indicated by white bars. Arrows mark binding sites of oligonucleotides used for construction of the deletion plasmid and for verification of the deletion. Flanking regions are shown as thin black bars and homologous integration is indicated by dotted lines. PCR fragments are indicated with black lines. (B) PCR analysis for verification of the *mek2* deletion strains S143932 and S1143995. Gene deletion was verified using primer pairs MEK2KO1/hph1MN, hph2MN/MEK2KO2 and gfp-mek2-fw/gfp-mek2-rv for amplification of the 5’ flank, 3’ flank and *mek2* gene, respectively. Wild type (wt) served as a control, where full length *mek2* was amplified. The negative control (NK) contained no genomic DNA. bp: base pairs.


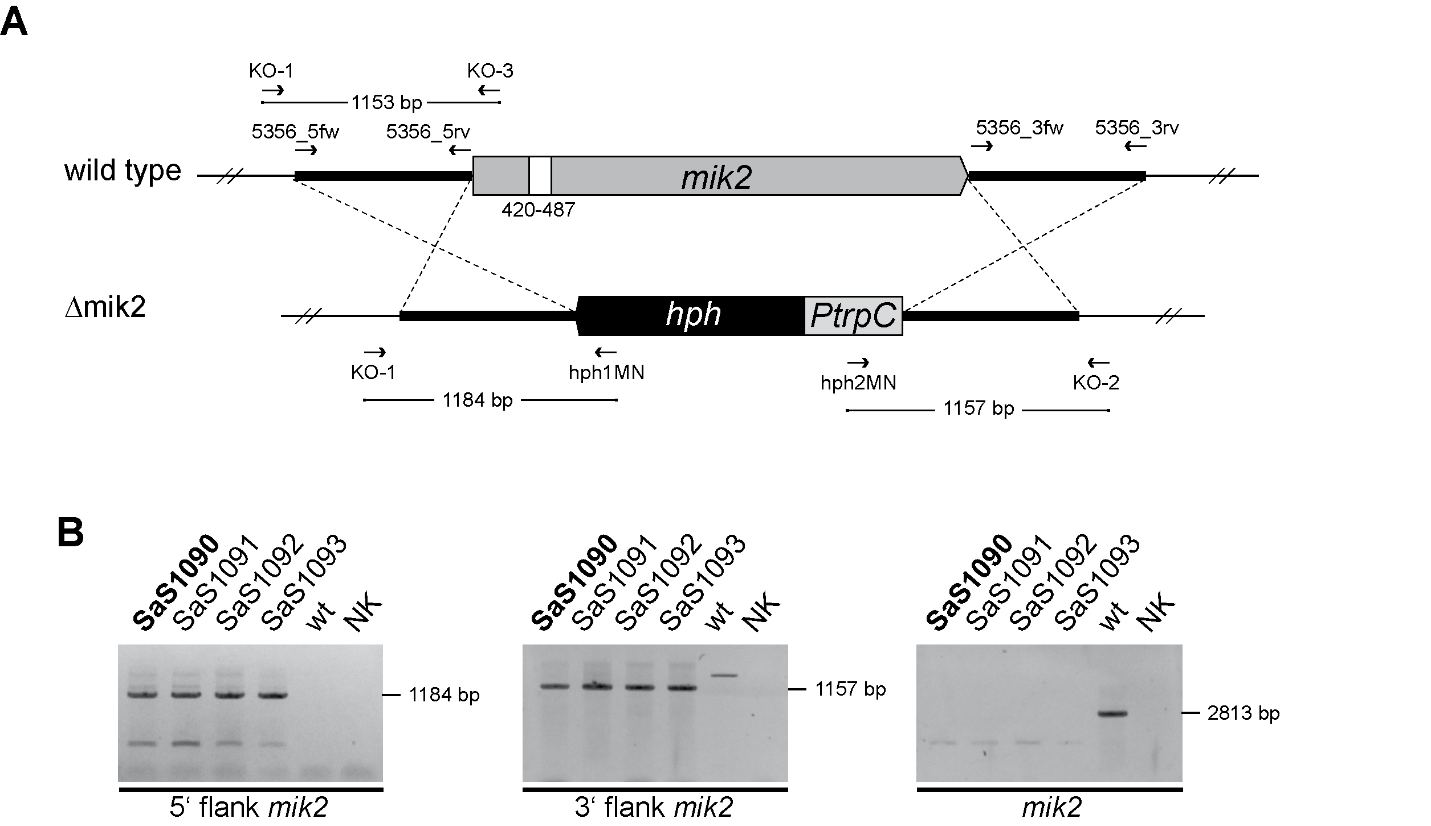


**Figure S3:** Generation and verification of Δmik2 deletion strains. (A) Genomic situation of the *mik2* locus in wild type (wt) and in the Δmik2 strain after homologous recombination. Introns are indicated by white bars. Arrows mark binding sites of oligonucleotides used for construction of the deletion plasmid and for verification of the deletion. PCR fragments are indicated with black lines. Flanking regions are shown as thin black bars and homologous integration is indicated by dotted lines. (B) PCR analysis for verification of the *mik2* deletion strains SaS1090, SaS1091, SaS1092 and SaS1093. Gene deletion was verified using primer pairs KO-1/hph1MN, hph2MN/KO-2 and KO-1/KO-3 for amplification of the 5’ flank, 3’ flank and *mik2* gene, respectively. Wild type (wt) served as a control, where full length *mek2* was amplified. The negative control (NK) contained no genomic DNA. bp: base pairs.


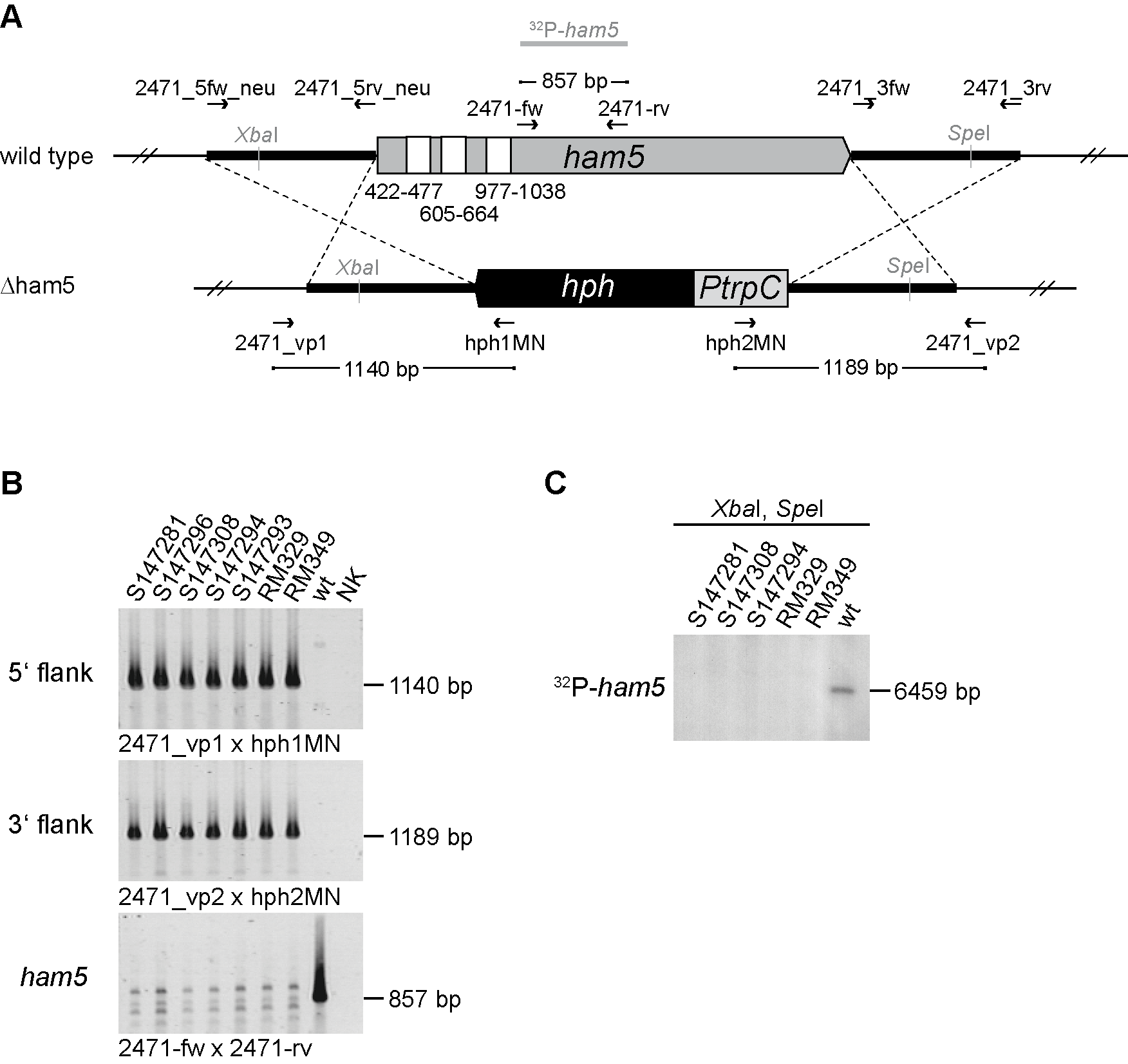


**Figure S4:** Generation and verification of ∆ham5 deletion strains. (A) Genomic situation of the *ham5* locus in wild type (wt) and the ∆ham5 deletion strain. Arrows mark oligonucleotides used for construction of the deletion plasmid and for verification of the deletion, and the black lines indicate the related DNA fragments amplified by PCR. Restriction enzyme sites and the probe used for Southern Blot analyses are indicated by grey letters and thick grey lines, respectively. (B) PCR analyses for verifications of the ∆ham5 deletion strains RM329, RM349, S147281, S147293, S147294, S147296 and S147308. The homologous integration of the 5’ and 3’ flank as well as the *ham5* deletion were verified by PCR using the indicated oligonucleotides. Wild type served as control for gene verification and negative control (NK) contained no genomic DNA. (C) Southern hybridization with the radioactively labelled probes specific for *ham5* after *Xba*I and *Spe*I hydrolysis of genomic DNA using the putative deletion strains RM329, RM349, S147281, S147294, S147308 as well as the wild type control. *hph*, hygromycin B resistance gene from *E. coli*; PtrpC, constitutive *trpC* promotor from *Aspergillus nidulans*.


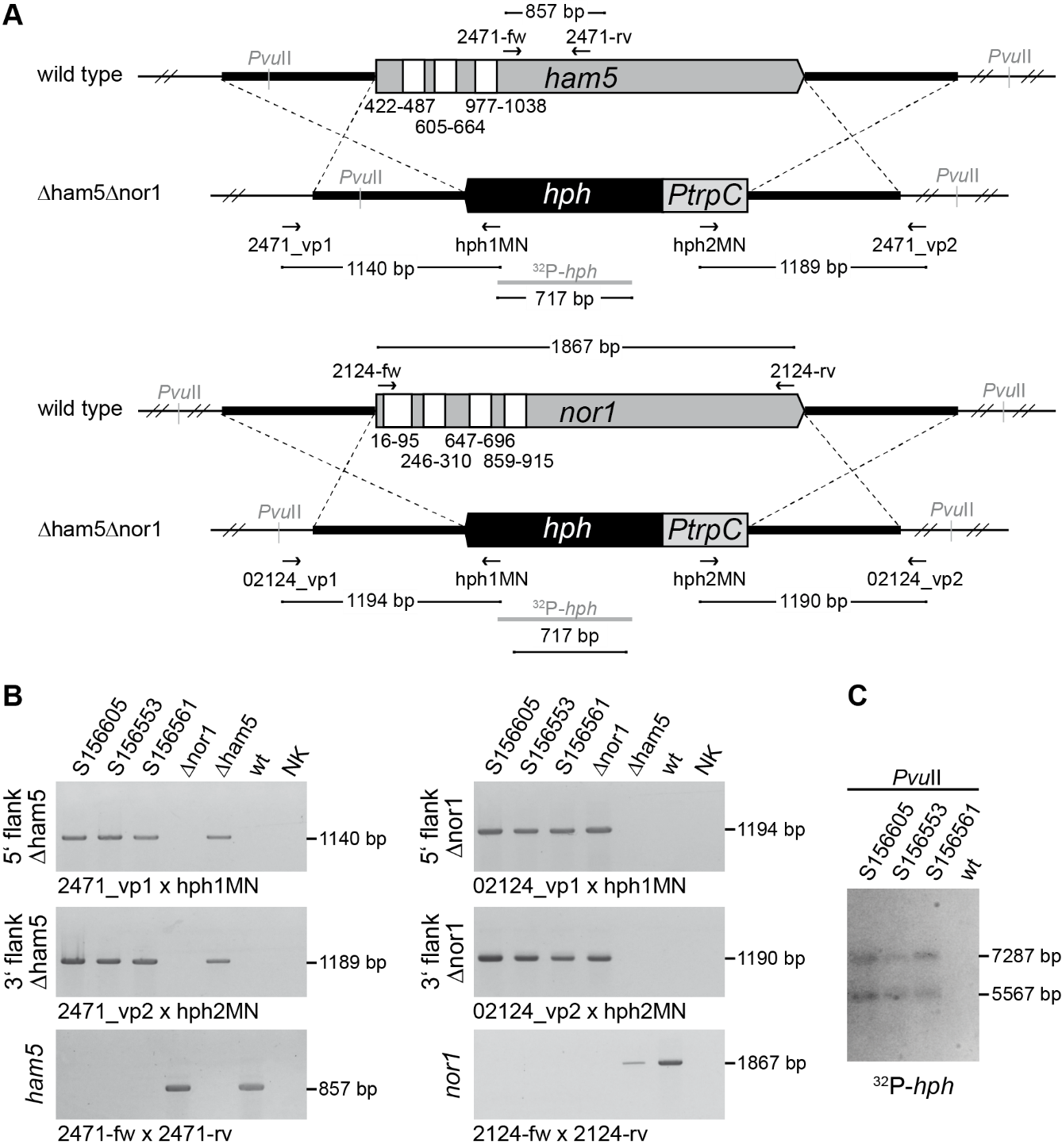


**Figure S5:** Generation and verification of ∆ham5∆nor1 deletion strains. (A) Genomic situation of the *ham5* and *nor1* loci in wild type and the ∆ham5∆nor1 deletion strain. Arrows mark oligonucleotides used for verification of deletions, and the black lines indicate the related DNA fragments amplified by PCR. Restriction enzyme sites and the probe used for Southern Blot analysis are indicated by grey letters and thick grey lines, respectively. (B) PCR analyses for verifications of the ∆ham5∆nor1 double deletion strains S156553, S156561 and S156605. The homologous integration of the related 5’ and 3’ flanks as well as the *ham5* and *nor1* deletions were verified by PCR using the indicated oligonucleotides. Wild type served as control for gene verification and negative control (NK) contained no genomic DNA. (C) Southern hybridization with the radioactively labelled probe specific for *hph* after *Pvu*II hydrolysis of genomic DNA using the putative deletion strains S156553, S156561 and S156605 as well as the wild type as control. *hph*, hygromycin B resistance gene from *E. coli*; PtrpC, constitutive *trpC* promotor from *Aspergillus nidulans*.


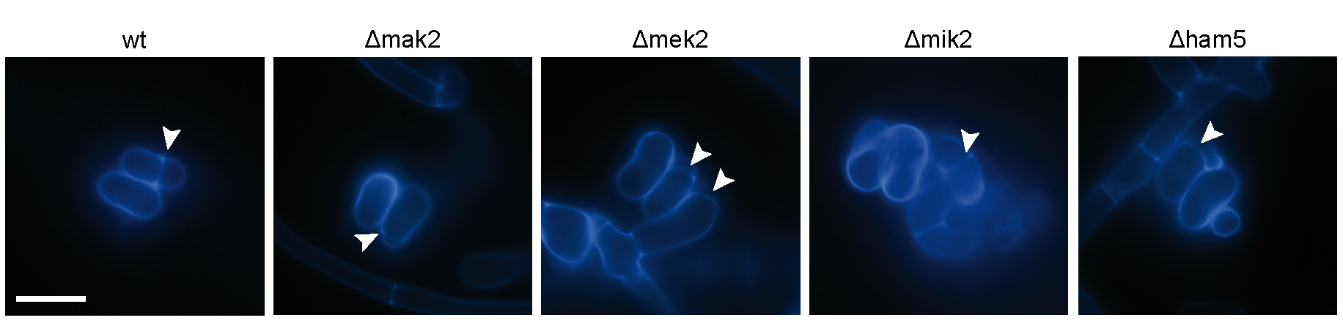


**Figure S6:** Ascogonial septation in Δmak2, Δmek2, Δmik2 and Δham5. Ascogonial coils were investigated for septation using CFW staining for visualization of the cell wall. Arrowheads indicate sites of ascogonial septation. Strains were grown on BMM-covered slides for 2 days, and 2.5 ng/ml CFW in 0.9% NaCl solution was applied before microscopic investigation. Scale bars indicate 10 μm.


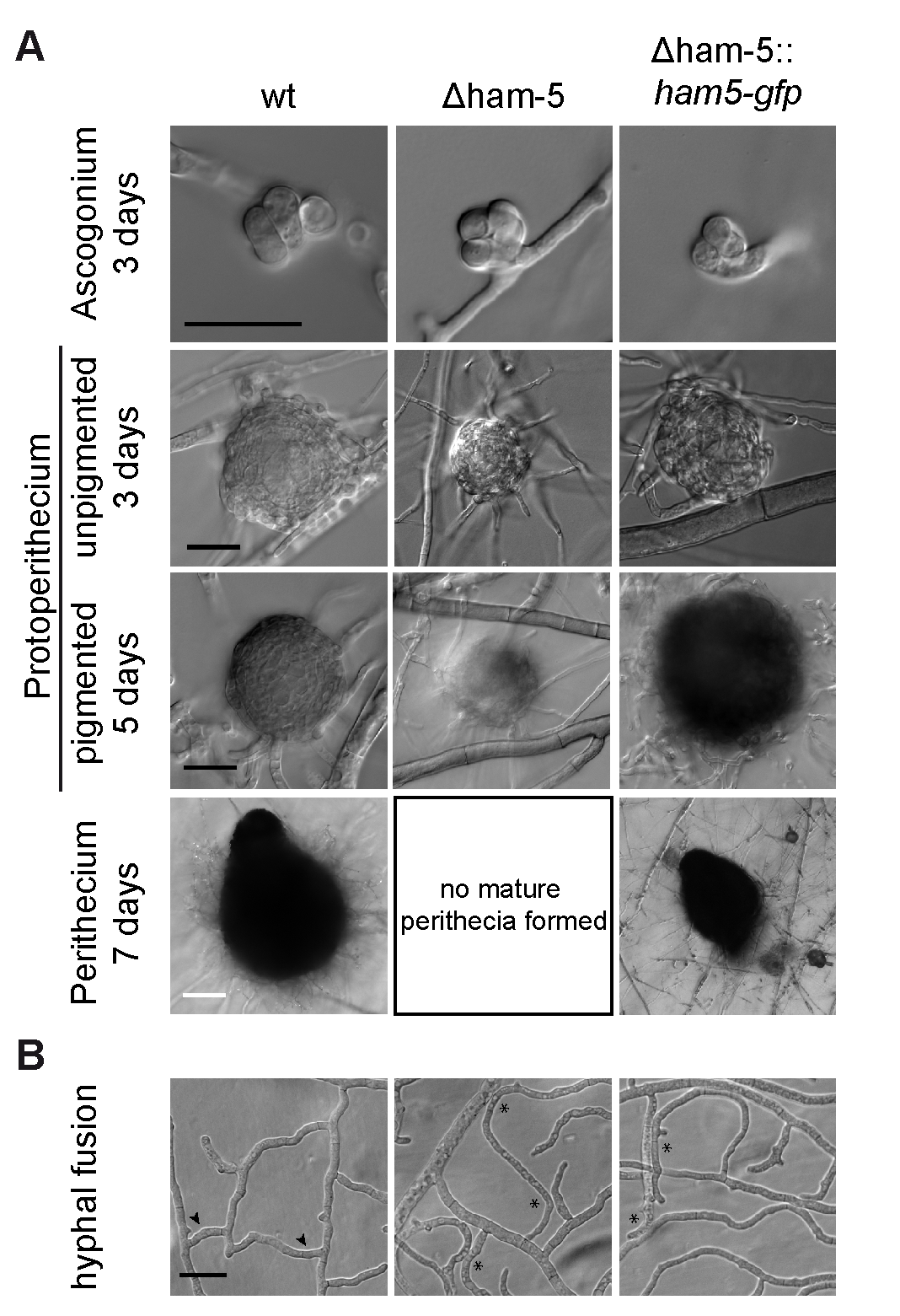


**Figure S7:** Characterization of Δham5 and Δham5::*ham5-gfp* strains. (A) Sexual development of strains grown for 3 to 7 days on BMM-coated slides. (B) 2 days after inoculation, hyphal fusion was analyzed on cellophane-covered solid MMS. Arrowheads indicate hyphal fusion events and asterisks mark hyphal contacts without fusion. The scale bar indicates 20 µm (black) and 100 µm (white). wt, wild type.


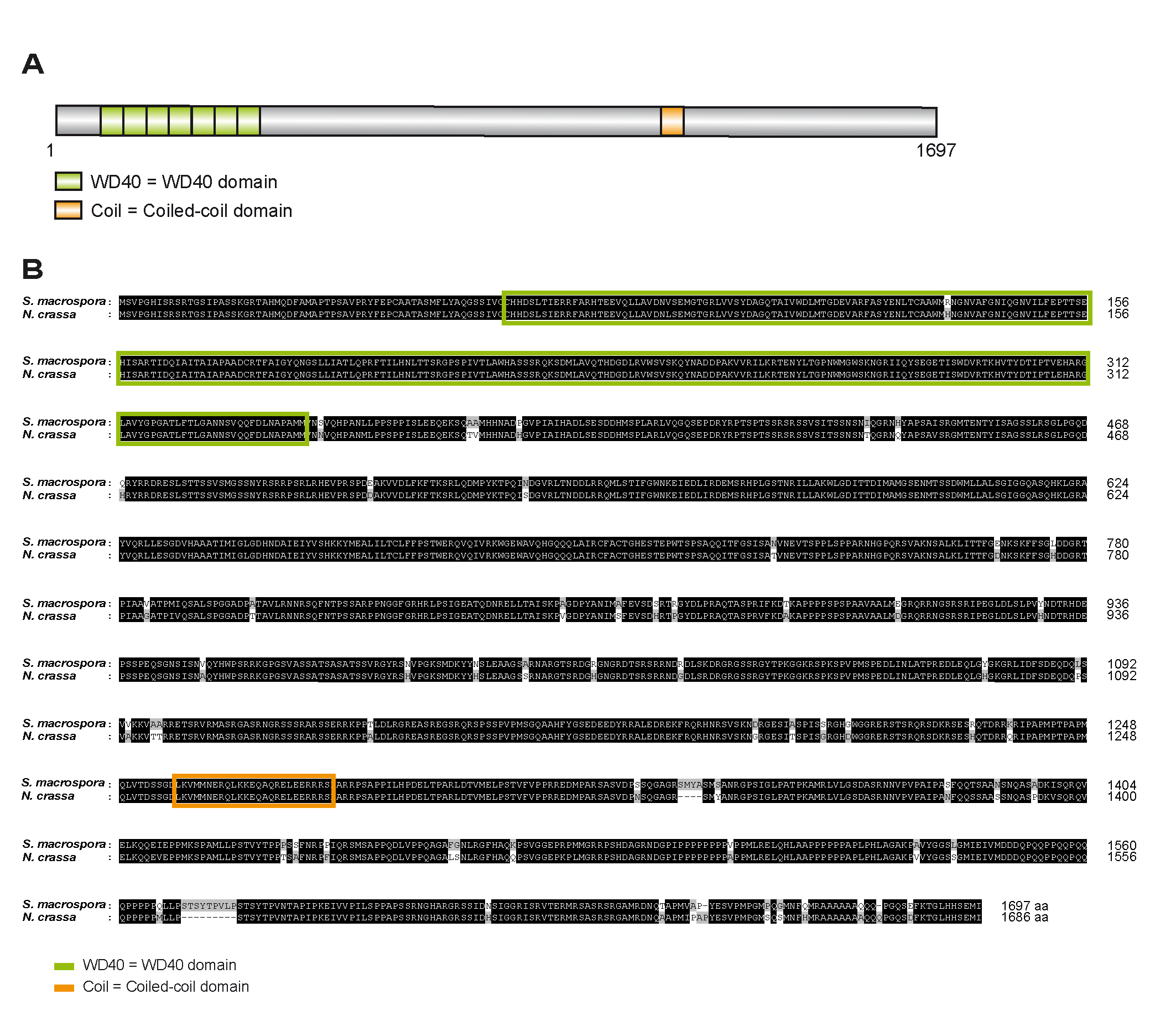


**Figure S8: Protein structure of HAM5.** (A) Protein domains of *S. macrospora* HAM5 were predicted *in silico* using the InterProScan (https://www.ebi.ac.uk/interpro/). (B) Alignment of the protein sequence of HAM5 from *S. macrospora* and its homologue from *N. crassa*.


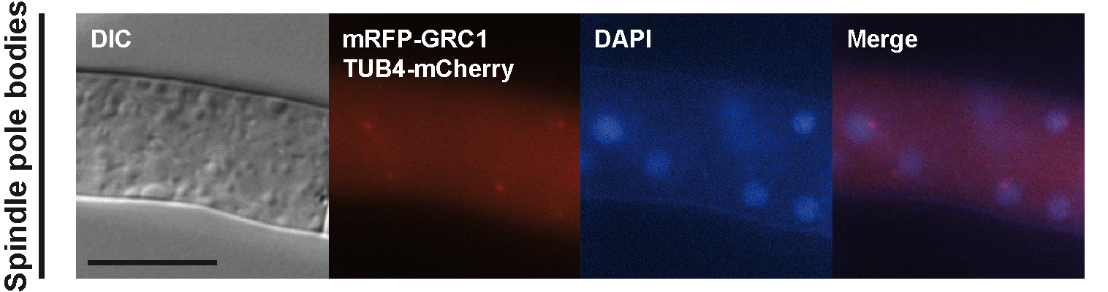


**Figure S9:** Fluorescent imaging of the SPB marker. Images of a wild type strain carrying the SPB marker consisting of mRFP-GRC1 and TUB4-mCherry. Nuclear association of SPBs was verified by counterstaining with DAPI. Images were obtained by epifluorescence microscopy. The scale bar indicates 10 μm. DIC: differential interference contrast.

**
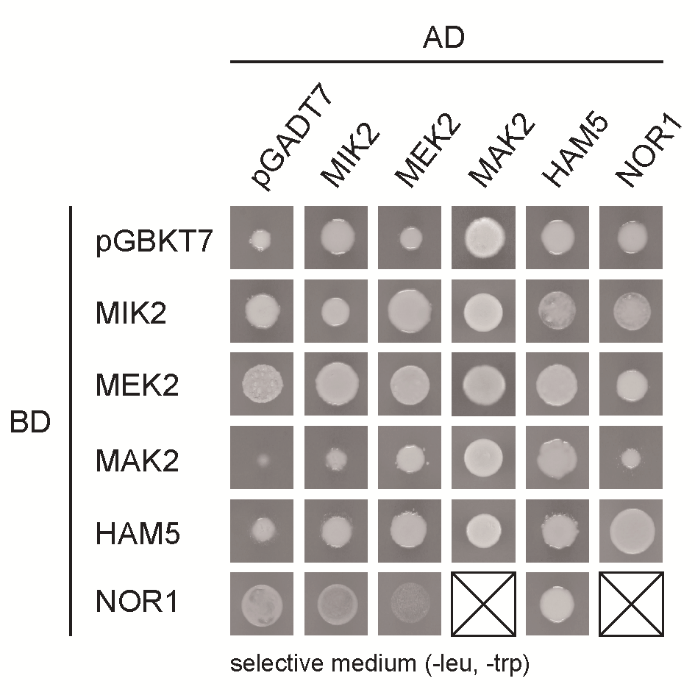
**

**Figure S10:** Growth control for yeast strains in Y2H analyses. Figure is related to Figure 4. Diploid strains were tested for growth on SD medium lacking leucine and tryptophan.
